# Proteomic Signatures of Epigenetic Age in African Green Monkey Cerebrospinal Fluid and Plasma

**DOI:** 10.1111/acel.70168

**Published:** 2025-07-29

**Authors:** John D. Elsworth, Albert Neutzner, Julien Roux, Juozas Gordevicius, Milda Milciute, Loveness Dzikiti, Dustin R. Wakeman, Brooke Danielsson, Cavit Agca, Matthew S. Lawrence

**Affiliations:** ^1^ Virscio Inc New Haven Connecticut USA; ^2^ Department of Biomedicine University of Basel Basel Switzerland; ^3^ Swiss Institute of Bioinformatics Basel Switzerland; ^4^ Epigenetic Clock Development Foundation Torrance California USA

**Keywords:** aging, blood, CSF, epigenetics, nonhuman primate, proteomics, vervet monkey

## Abstract

Strategies to slow the aging process or mitigate its consequences on health rely on the validation of minimally‐invasive biomarkers of aging that can be used to track aging and test the effectiveness of antiaging interventions. Study of aging in a nonhuman primate species offers a robust translational approach to achieving these aims, avoiding wide differences in genetics and environmental exposures that confound human aging studies. As epigenetic age appears to predict biological aging, biomarkers linked to epigenetic aging should be especially valuable in identifying individual differences in aging progression and documenting the impact of antiaging strategies. Proteins are the final effectors in most signaling pathways, indicating that alteration in levels of circulating proteins potentially offers an informative and valuable quantitative index of aging. Accordingly, a proteomic analysis was conducted on matching CSF and plasma samples collected from a large group of African green monkeys, with epigenetic ages ranging from young to old as determined by differential methylation of blood DNA. In addition to analyzing the data with linear statistical models, a gradient boosting machine learning technique was employed to identify not only individual CSF and plasma proteins that correlated with aging progression but also groups of proteins that could be used as predictors of global aging and of specialized aspects of aging such as inflammation. Overall, this study identified new CSF and plasma protein targets for understanding aging biology, together with identifying biomarkers to track changes in the rate of biological aging in a translationally relevant nonhuman primate model.

## Graphical abstract

Epigenetic age together with plasma and CSF proteomes were quantified in a large number of African green monkeys. This study identified new accessible protein biomarkers (“clocks”) for understanding aging biology and to track changes in the rate of biological aging in a translationally relevant nonhuman primate model.

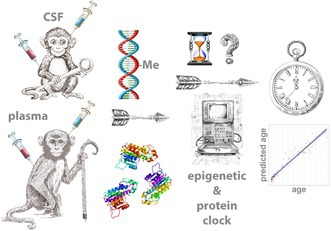

## 1 Introduction

Advancing age is the major risk factor and predictor for numerous prevalent diseases (Kennedy et al. 2014). With a global aging population, there is an urgent need both to gain a deeper understanding of the aging process and to develop reliable methods to test antiaging strategies. Progress in understanding and testing is necessary for improved treatment of age‐associated disease and effective interventions to increase healthspan (Campisi et al. 2019).

There are differences among individuals between their chronological age—the time since birth—and biological age, defined as an individual's age established by the level of age‐dependent biological changes, such as accumulation of molecular and cellular damage (Moqri et al. 2023). The aging process has been characterized by multiple interconnected cellular and molecular hallmarks (Lopez‐Otin et al. 2023). Consequently, a major challenge in aging research is the development of biomarkers that could accurately track the biological age of an individual and discriminate, at an early stage, different health trajectories (Aging Biomarker Consortium 2023; Galkin et al. 2020). Success in this approach would further our understanding of the aging process and facilitate the design of strategies that prevent, delay, or reduce the severity of age‐related diseases, inform clinical trial interpretation, and enable more accurate epidemiology and effective public health policy.

Alterations in protein levels in easily accessible tissues, such as blood, have long been known to be reliable indicators of certain disorders, such as cardiovascular disease (Ellis 1991) and to alter during normal aging (Bruunsgaard et al. 2000). With the ability to quantify the abundance of over a thousand proteins, recent proteomics studies in humans have identified many proteins whose abundance increases or decreases in the blood with age (Argentieri et al. 2024; Coenen et al. 2023; Goeminne et al. 2025; Johnson et al. 2020; Kuo et al. 2024; Oh et al. 2023; Sathyan et al. 2020). Validation of proteins that best reflect the aging process is hampered by variation in health, genetics, lifestyle, and environmental and pharmacological exposures across human individuals, although some studies have been conducted using large well‐phenotyped cohorts (e.g., UK Biobank database). In addition, methodological differences are present across studies, impacting the panels of assayed proteins and their quantification. Nevertheless, because proteins are the final effectors of most biochemical processes, probing the proteome for signatures of aging holds great promise for understanding the aging process, defining enhanced aging biomarkers, and identifying targets for antiaging interventions.

Humans share great similarity in lifespan, genomics, biochemistry, and pharmacological response to therapeutics with nonhuman primates (NHPs) (Phillips et al. 2014). Consequently, studies conducted in NHPs are highly translationally relevant and can be more tightly controlled than studies on humans. Identification of validated NHP aging biomarkers would allow their application as endpoints in future NHP studies of aging biology and antiaging interventions. The current study reports changes in the proteome across the lifespan of African green monkeys (*Chlorocebus aethiops sabaeus*) using a proxy of biological age as the independent variable. Quantification and analysis of proteins in matched plasma and CSF samples enabled a novel comparison of changes in the peripheral and central compartments.

The St Kitts origin African green monkey is one of the most studied nonhuman Old World primates, with extensive genomic and systems characterization, constrained environmental and genetic variability, low infectious disease burden, and a long history of use in biomedical research supporting preclinical drug development (Jasinska et al. 2013, 2017). The animals sampled in the present study were housed at the St Kitts Biomedical Research Foundation (SKBRF), a facility in St Kitts, an island in the West Indies, and are derived from a small founder population introduced from West Africa about 350 years ago. Some of the African green monkeys at the facility originate from the wild population on the island, and the birthdate of such animals is unknown, so we used an epigenetic “clock” (Horvath and Raj 2018) based on changes in CpG DNA methylation at discrete sites in the genome as a proxy for biological age. Epigenetic clocks have been used to identify differences in biological aging that skew individuals of a given chronological age from their expected epigenetic age (Horvath and Raj 2018; Wang et al. 2022). Accelerated epigenetic aging has been associated with many diseases and even predicts all‐cause mortality risk, independent of chronological age, lifestyle habits, and morbidity (Colicino et al. 2020). Using a large group of male and female African green monkeys, we identified proteins in their plasma and CSF significantly associated with epigenetic age. These novel biomarkers in African green monkeys should prove useful in studies of biological aging mechanisms and anti‐aging interventions.

## 2 Methods

### 2.1 Animals

African green (“vervet”) monkeys (*Chlorocebus aethiops sabaeus*) were housed at SKBRF, an organization accredited by AAALAC International, and were either born at the facility or were sourced from the abundant free‐ranging population located on the island of St. Kitts. The lifespan of African green monkeys is typically 20–30 years old in a protected and enriched environment such as the outdoor social group enclosures of SKBRF. Blood and cisternal CSF samples were drawn from the animals while sedated by an intramuscular injection of ketamine (8 mg/kg) and xylazine (1.6 mg/kg).

### 2.2 Epigenetic Blood Clock

Venous blood samples were collected from 96 male and female African green monkeys with known chronological ages ranging between from 3 months to 17 years old. Samples (0.5 mL) were gently mixed in K_2_EDTA tubes to prevent coagulation and 0.1 mL aliquots transferred to screw‐cap microtubes (Sarstedt, item 72.730.005) and stored in a −80°C freezer. DNA was extracted from a single aliquot from each monkey using a spin‐column protocol “DNeasy Blood & Tissue” kit (Qiagen, item 69506). Final eluates were further purified by the “DNA Clean and Concentrator‐5” kit (Zymo Research, item D4014). A microvolume spectrophotometer (NanoDrop One, Thermo Fisher Scientific) determined the concentration (≥ 15 ng/μL) and purity (mean ± S.D., 260/280 = 1.86 ± 0.04, 260/230 = 2.30 ± 0.11) of isolated DNA and the final extract (~50 μL) was stored in “Safe‐Lock” microtubes tubes (Eppendorf, item 0030123603) within a −80°C freezer. A portion (30 μL) of each extract was transferred to a 96‐well plate, which was sealed and shipped in a liquid nitrogen vapor shipper to Virscio laboratories (New Haven, CT) and stored in a −80°C freezer until delivery on dry ice to the Epigenetic Clock Development Foundation (clockfoundation.org) for analysis of DNA methylation sites.

### 2.3 DNA Methylation Profiling

The isolated DNA underwent bisulfite conversion using the Zymo EZ DNA methylation kit (Zymo, Irvine, CA, USA). A minimum of 150 ng of total bisulfite‐converted DNA was processed using the Horvath 320 k methylation assay (Epigenetic Clock Development Foundation, Los Angeles, CA, USA). DNA methylation was measured using the custom Illumina chip “HorvathMammalMethylChip40”. Raw data were normalized using the Sensible Step‐wise Analysis of Methylation data (SeSAMe) R package (Zhou et al. 2018), resulting in a methylation estimate (beta value) corresponding to each array probe for every individual in the dataset and a detection *p* value corresponding to the confidence in the normalized beta value. Beta values from SeSAMe were derived from the ratio of the fluorescence intensity of a methylated probe for a specific CpG to the total overall probe intensity (the sum of the signal from both the methylated and unmethylated probes plus a constant) (Du et al. 2010). Beta values range from zero to one, with a value of zero indicating that no copies of the gene were methylated. To identify technical outliers after SeSAMe normalization, an unsupervised hierarchical clustering analysis based on the inter‐array correlation was used. Data were filtered by detection *p* value as calculated in SeSAMe (Zhou et al. 2018).

### 2.4 Statistics: Epigenetic Age Determination

To construct the reliable and accurate epigenetic clock, DNA methylation data from the Virscio African green monkey blood clock was integrated with data from a blood clock developed using the African green monkey colony at the Vervet Research Colony (VRC, Wake Forest). This latter colony was originally established 4 decades ago with 57 founder monkeys imported from St Kitts (Jasinska et al. 2022). Additional internal datasets from Virscio's African green monkey blood samples were also included, resulting in a combined dataset of 336 samples. These samples were processed as previously described, with unreliable probes filtered out if detection *p* values exceeded 0.05 in more than 90% of the samples. To construct an epigenetic clock, the dataset was split into training (70%) and validation (30%) subsets. The preProcess function from the R package caret was used to remove variables with zero or near‐zero variance, while also centering and scaling the beta value matrix. A 10‐fold cross‐validation procedure was employed to train an age predictor using the glmnet method, which implements elastic net regularization. The algorithm tested 10 different lambda values to identify the optimal parameter that minimized the root mean squared error. The resulting clock was validated on the independent validation dataset and subsequently used for determining the epigenetic age of animals of unknown chronological age used in the proteomic study reported here.

### 2.5 Sample Collection for Proteomic Analysis

Samples of venous blood and cisternal CSF were collected from 25 male and 74 female African green monkeys of unknown chronological age, estimated to range between 4 and 25 years old based on physical characteristics and behavior (Wakeman et al. 2022). Animals were food fasted overnight before collections that took place between 9 am and 1 pm; specifically occurring between 9 and 10 am for 12%, between 10 and 11 am for 66%, between 11 am and noon for 19%, and between noon and 1 pm for 3%. Each CSF sample was divided among cryotubes and quickly stored within a −80°C freezer. Each blood sample was added to a lithium heparin vacutainer tube, gently mixed, stored on ice, and within 1 h for the majority and 2 h for all samples, centrifuged for 10 min at 1300 × g at 4°C to separate plasma, which was removed and quickly stored in cryotubes within a −80°C freezer. Plasma and CSF samples were shipped in a liquid nitrogen vapor shipper to Virscio laboratories (New Haven, CT) and stored in a −80°C freezer until delivery on dry ice to Olink Proteomics (https://olink.com) for analysis on the “Olink Explore 1536” platform, a multiplex proximity ligation immunoassay allowing the detection and quantification of 1463 unique proteins in each sample. The time between sample freezing at SKBRF and first thawing of samples immediately before analysis at Olink was between 7 and 14 months. The collection and storage conditions used are in accordance with data from Olink regarding optimal sample processing for proteomics (Shen et al. 2018). Sequence similarity of the assayed human proteins to their African green monkey orthologs was verified and was overall very high (Figure S1). Subsequently, after establishing the epigenetic blood clock for the African green monkey (described above), most of the animals (21 male and 60 female) used for the Olink proteomic analyses were resampled to collect another blood sample for extraction of DNA, as described above, for determination of epigenetic ages, which was used as the independent variable in the proteomic analyses.

### 2.6 Statistics: Protein Data Analyses

The dependent variable for the Olink protein assay is a normalized protein expression (NPX) value, which is an arbitrary relative quantification unit on a log2 scale, based on the number of cycles needed for the fluorescent signal to surpass a threshold line in the qPCR analysis for each protein (cycle threshold, Ct). A high NPX value corresponds to a high protein concentration, and because NPX is on a log2 scale, a 1 unit NPX increase represents a doubling of protein concentration. The Olink quality control consists of four internal controls that are spiked into every sample and are designed to monitor the three main steps of the Olink protocol: immunoreaction, extension, and amplification/detection. A principal component analysis was performed on the NPX values in CSF or plasma samples together and separately (R package stats, v4.4.1). A scatterplot of the first two principal components was examined to identify outlier samples; no sample was eliminated from data analyses.

The initial analyses to detect age‐dependent protein NPX values used an analysis of variance (ANOVA) with the Benjamini–Hochberg method to correct for multiple comparisons (Lualdi and Fasano 2019). Proteins that passed an adjusted *p* value threshold of 0.05 were then used in post hoc testing of pairwise differences in abundance among 3 predefined epigenetic age groups. The chosen age bins were chosen to reflect developmental timelines for this species and to balance sample sizes within each bin (Figure S2A). Four years of age marks the approximate onset of sexual maturity, so the 4–11 years group corresponds to young adults (Y), with the 12–19 years group representing middle‐age (M), with animals ≥ 19 years being referred to as aged (A). In addition, a linear regression analysis was performed (epigenetic age vs. NPX value) for each protein that exhibited significant changes across the 3 age bins to determine the strength of the association between individual protein abundance and age in CSF and in plasma.

Subsequently, the limma R/Bioconductor package (3.60.6) was used to identify differential expression of proteins according to age after loess normalizing of NPX values (Ritchie et al. 2015). Later on, machine learning techniques including gradient boosting (R package XGBoost v1.7.8.1) and k‐means clustering (R package stats v4.4.1) were used (Bock 2008; Chen and Guestrin 2016). It was anticipated that these algorithms would reveal more complex relationships in the data than ANOVA and would help in analyzing, interpreting, and visualizing the proteomic data.

### 2.7 Statistics: Summary

The main goals of the study were (1) to identify novel proteins in plasma and CSF that change in abundance during epigenetic aging, (2) to discern how accurately a small subset of specific CSF or plasma proteins could predict epigenetic age (“protein clocks”), and (3) to identify specific cellular pathways affected by epigenetic aging, using Ingenuity Pathway Analysis (Kramer et al. 2014).

## 3 Results

### 3.1 African Green Monkey Blood Clock

**FIGURE 1:**
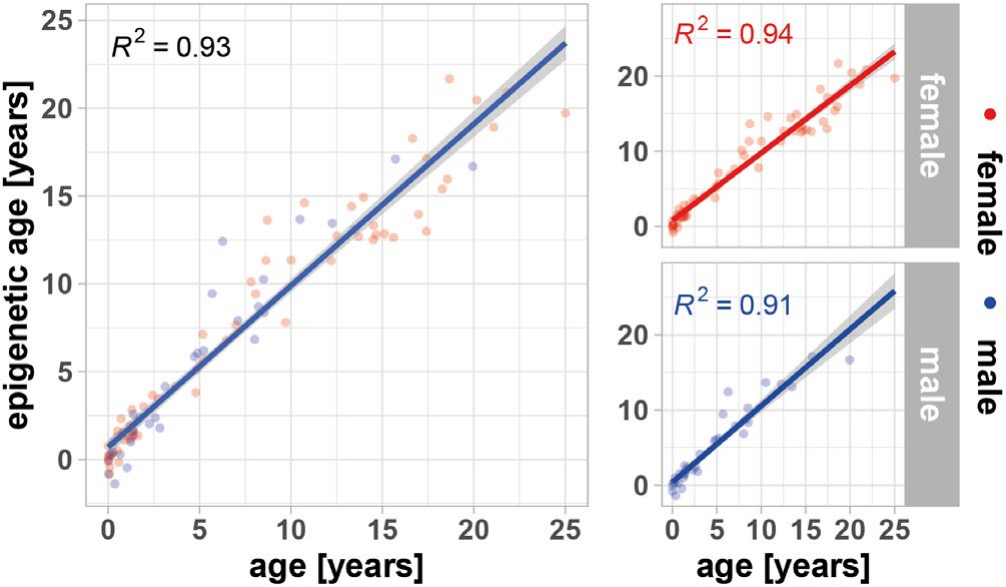
Establishing an African green monkey epigenetic blood clock. DNA methylation analysis was conducted on blood samples from 336 animals of known chronological age; 70% were used for model construction and remaining 100 samples were used for the shown validation. The 100 animals comprised both female and males with mean age: 6.5 ± 6.7 years; 64 females with mean age: 7.5 ± 7.3 years; 35 males with mean age: 4.5 ± 2.4 years. The figure shows a linear regression of chronological versus epigenetic age with annotation of the coefficient of determination (*R* ^2^).

Based on DNA methylation patterns, an epigenetic clock was constructed using DNA extracted from the blood of St Kitts African green monkeys having a known birth date. The final model was optimized with a lambda value of 0.98, which minimized the root mean squared error (RMSE) during the 10‐fold cross‐validation process. This lambda value was used in the final epigenetic clock model, which was validated on the independent validation dataset. The resulting model was able to predict epigenetic age with high confidence (correlation coefficient (*r*) 0.97, coefficient of determination (*R* ^2^) 0.93, and a mean absolute error (MAE) of 1.2 years). This clock was then used to derive the epigenetic age of each monkey in the proteomic study of aging (Figure 1). The median epigenetic clock age of animals in the study was 18 ± 4.4 years.

### 3.2 Initial Analysis of Proteomic Data

**FIGURE 2:**
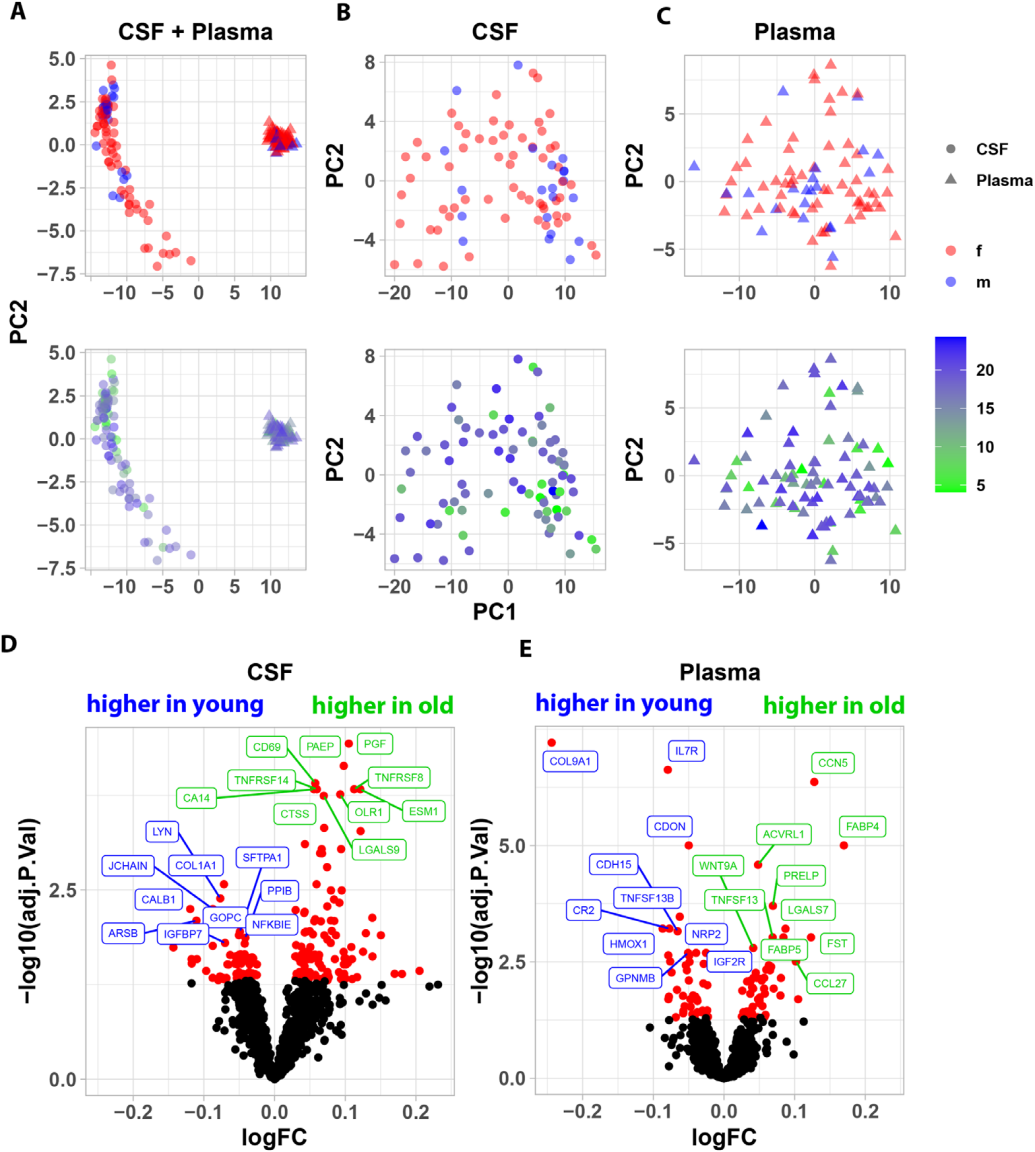
Cluster analysis and determination of differentially expressed proteins. Principal component analyses for unsupervised sample clustering were performed based on top 150 (A) CSF and plasma, (B) CSF, (C) plasma loess normalized NPX values, showing distribution based on either sex (upper panel of A, B, C) or epigenetic years (lower panels of A, B, C). In 2A–C, CSF samples are depicted as circles and plasma as triangles. Using limma with epigenetic age as continuous variable, differences in (D) CSF and (E) plasma protein levels between young and aged animals were analyzed.

The levels of 1463 CSF and plasma proteins were determined in 81 animals and expressed as NPX values (Figure S2B). We performed principal component analysis (PCA) based on loess normalized CSF and plasma protein levels (Figure 2A) and observed a clear separation between plasma and CSF samples on the first principal component, explaining 87.8% of the observed variance (Figure S3). When performing PCA on CSF (Figure 2B) or plasma (Figure 2C) samples separately, no obvious clustering of samples was detected, neither based on sex nor epigenetic age.

We used an ANOVA with post hoc testing to identify proteins that exhibited progressive significant changes across 3 predefined age groups (i.e., Y > M > A or Y < M < A; Table S1A). In addition to the two proteins displaying such a pattern in CSF, there were 47 additional CSF proteins and 100 plasma proteins that had significant changes in protein abundance across 2 of 3 age group combinations (e.g., Y < M, Y < A, and Y ≮ M); eight of these proteins were in both CSF and plasma (ACVRL1, COL1A1, CST1, EDA2R, FGFR2, KLK6, OXT, and TNFRSF19). The 10 CSF and 10 plasma proteins that demonstrated the highest degrees of significance are in Table S1B. In addition, to examine to what extent age contributed to the variance in abundance for individual proteins in Table S1, linear regression analyses were performed, with statistical results shown in those tables. A two‐way analysis of variance (ANOVA) model using age and sex as independent variables was run to assess which proteins exhibited male–female differences in the levels of age‐dependent proteins or a sex*age interaction. This indicated that there was a significant main effect of sex for no proteins in CSF and 44 plasma proteins and a sex*age interaction for no proteins in CSF and 2 plasma (GNE and PPCDC) proteins; however, none of these proteins were among those identified by later analyses as relevant for age prediction.

### 3.3 Unbiased Determination of Age‐Related Changes in CSF and Plasma Proteins

Using limma with a model including age as a continuous variable, we identified 188 CSF and 99 plasma proteins significantly associated with age (FDR < 0.05; Figure 2D,E). However, the size of age‐dependent changes was low (CSF: mean logFC = 0.025 ± 0.07; Plasma: mean logFC = 0.007 ± 0.06). To establish an alternative unbiased epigenetic age cutoff between young and aged animals, we performed k‐means clustering to partition sets of proteins into statistically separate clusters based on their correlation to epigenetic age. A preliminary examination of the distribution of correlations among the proteins revealed, as expected, that relatively few proteins (correlation greater than: number of proteins; ±0.4: 93; ±0.5: 15; ±0.6: 2) exhibited a strong correlation with age (Figure S4A). Cluster analysis based on proteins with an epigenetic age‐correlation coefficient of >±0.4 to ±0.6 grouped animals into two clusters with significantly different mean ages. The mean age of animals contained in the cluster of older animals was around 19 years (±0.4/±0.5: 18.8 ± 1.9 years; ±0.6: 18.75 ± 2.1 years), while the mean age of animals grouped into the cluster of younger animals varied between 9.2 ± 5.6 and 13.4 ± 7 years (Figure S4B).

Next, we performed PCA analysis using 93 proteins with a Spearman coefficient of > ±0.4. Confirming k‐means clustering, PCA resulted in a clear separation of animals into young (5–10 years) and aged animals (> 16 years) with intermediate aged animals (11–15 years) located between these two clusters (Figure S4C). We then explored whether epigenetic age can be inferred from the levels of age‐correlating proteins. For this, we fitted a linear model for each of the 93 best age‐correlated proteins (Spearman > ±0.4) on a training set generated by a 70:30 split (training:holdout—Figure S5) and predicted the epigenetic age of the holdout samples by averaging the predicted ages across the 93 proteins used. As shown in Figure S4D, calculation of epigenetic age for samples in the holdout data set did not yield accurate age prediction. While the age of older animals was underestimated, the age of younger animals was overestimated. We confirmed this through the analysis of residuals showing non‐normal distribution (Anderson‐Darling test: A = 0.76; *p* value = 0.04) indicative of insufficient modeling accuracy.

### 3.4 Identification of Age‐Predictive Proteins Through XGBoost Analysis

**FIGURE 3:**
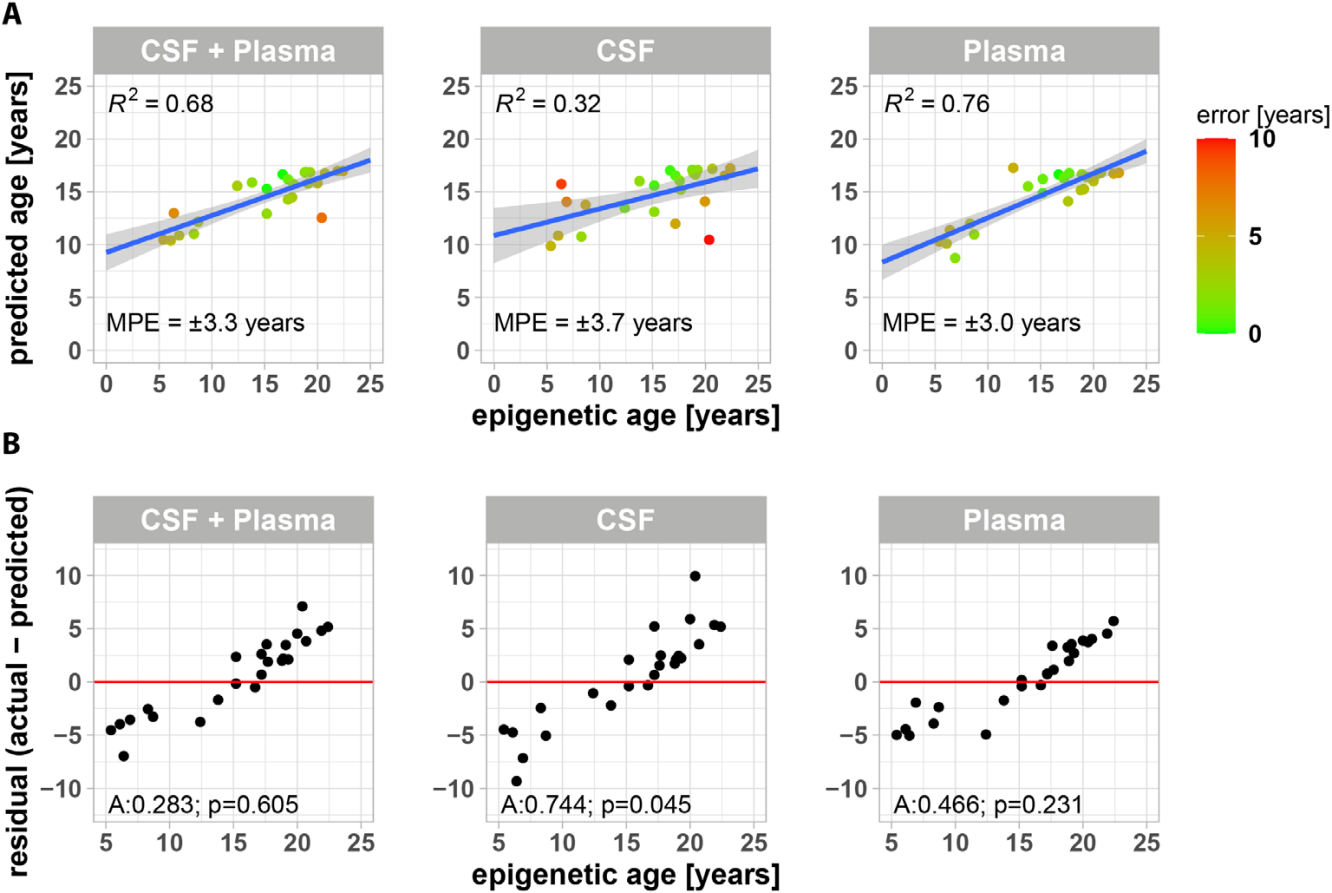
XGBoost modeling to predict epigenetic age from CSF and plasma NPX values. (A) A dataset of 1472 CSF and 1472 NPX plasma levels from 81 animals was randomly split 70:30 into a training and test dataset, respectively (see Figure S5). Hyperparameter‐optimized XGBoost models for regressing epigenetic age from CSF and/or plasma protein features were trained on the training dataset and epigenetic age of animals in the test dataset was predicted. Shown are linear models of epigenetic versus predicted age of animals in the test dataset with the respective coefficient of determination (*R* ^2^) and (B) Anderson‐Darling testing of residuals for normality as measure of model robustness. Annotation: MPE is the mean prediction error in years, A is the Anderson‐Darling statistics and its *p* value, with *p* > 0.05 signifying normal distribution of residuals.

To improve epigenetic age prediction, we used XGBoost regularizing gradient boosting to infer epigenetic age from plasma and CSF protein levels. For this, we randomly split the sample data from the 81 animals into a training (57 animals) and hold‐out test set (24 animals) while preserving age distribution (Figure S5). Following hyperparameter tuning, we generated XGBoost models based on plasma and/or CSF protein levels (Figure 3A). We found that hyperparameter tuned models trained on levels of all proteins (both in CSF and plasma) were able to predict epigenetic age from hold‐out data with a mean error of 3.3 years and an *R* ^2^ = 0.68. Models trained only on CSF or only on plasma protein levels were slightly less predictive with a mean error of 3.7 years (*R* ^2^ = 0.32) and 3.0 years (*R* ^2^ = 0.76), respectively. Analysis of residuals confirmed a reduction of the age underestimation for older animals and age overestimation for younger animals (Figure 3B).

**FIGURE 4:**
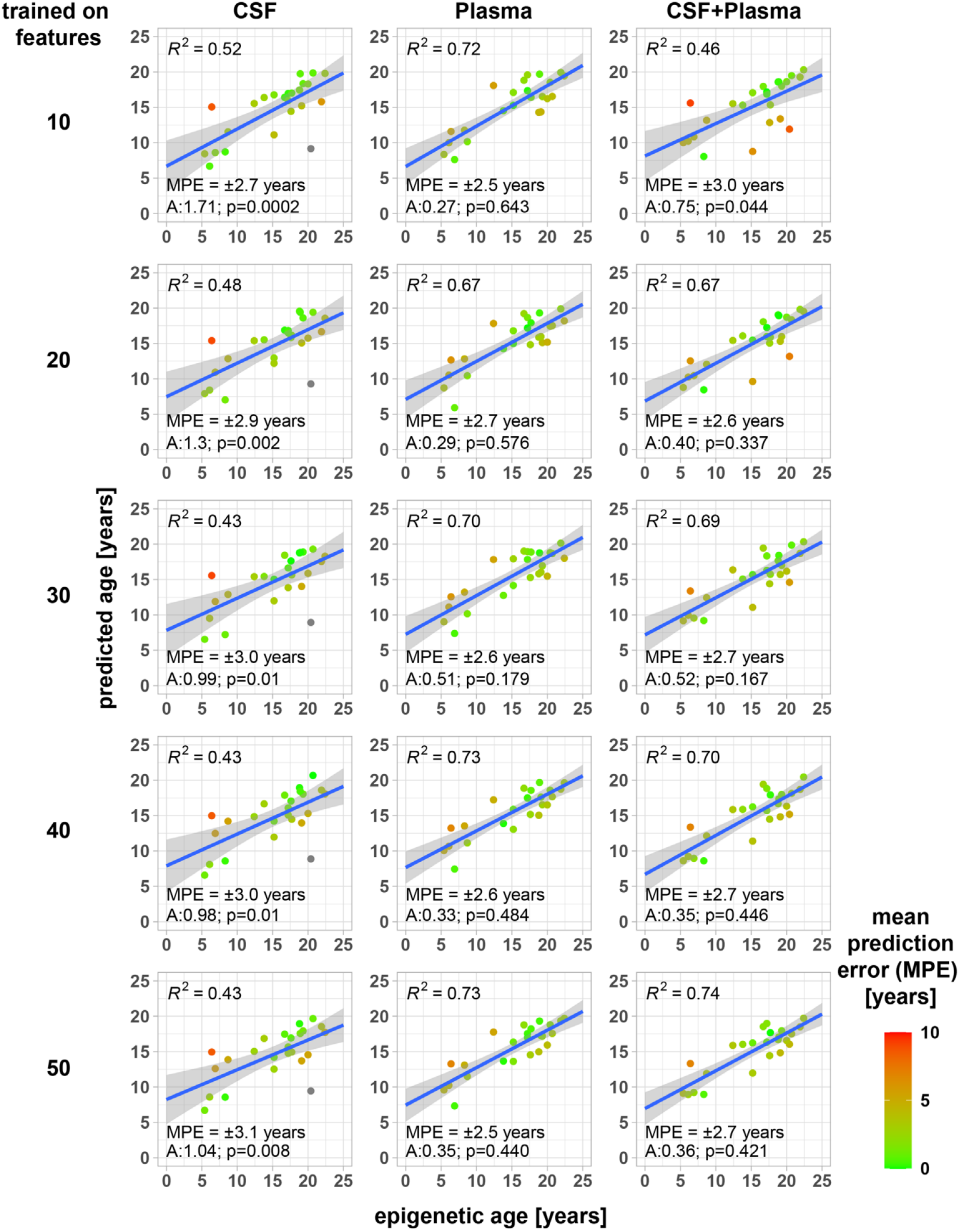
Determination of minimally effective XGBoost feature space. (A) Between 10 and 50 CSF and/or plasma protein features identified as high gain in XGBoost models trained on complete datasets of 1472 CSF and/or plasma protein features (compare to Figure 3) were used for XGBoost model hyperparameter optimization. Based on the best performing hyperparameter set (compare to Figure S7), XGBoost models were trained on the 70:30 training dataset (Figure S5) and used to regress epigenetic age of the test data set with the boxplot. Plots are annotated with the R^2^ of the resulting linear regressions, the mean prediction error (MPE) in years, and the Anderson‐Darling test for residual distribution analysis (A and p).

In a next step, we aimed at minimizing the feature space for XGBoost model training, while retaining predictive power, in order to identify a reduced set of proteins for epigenetic age prediction. For this, we selected 50 high‐gain protein features (CSF + plasma, CSF, plasma) that contributed to the predictive power of the above‐described XGBoost models (Figure S6). Using hyperparameter tuning (Figure S7), we trained additional XGBoost models on decreasing numbers of features and evaluated their performance by repeatedly predicting epigenetic age and determining *R* ^2^ of the resulting predicted vs. epigenetic age linear model (Figure 4). Interestingly, we found that models trained on only 10 plasma protein features had predictive power comparable to models trained on complete feature sets (*R* ^2^ for linear fit of predicted vs. known epigenetic age: Top 10 plasma—*R* ^2^: 0.72). Similarly, 10 protein features were sufficient to generate XGBoost models with high predictive power for CSF (*R* ^2^: 0.52) and for CSF + plasma (*R* ^2^: 0.46). As shown in Figure 4, XGBoost models based on 10 highest‐gain CSF and/or plasma features and above established hyperparameters predicted epigenetic age with ±2.5 (plasma), ±2.7 (CSF), and ±3.0 (CSF + plasma) years with normal residual distribution. Thus, limiting the feature space did not negatively impact model performance.

### 3.5 Comparing Age‐Correlating Proteins With XGBoost Protein Feature Set

**FIGURE 5:**
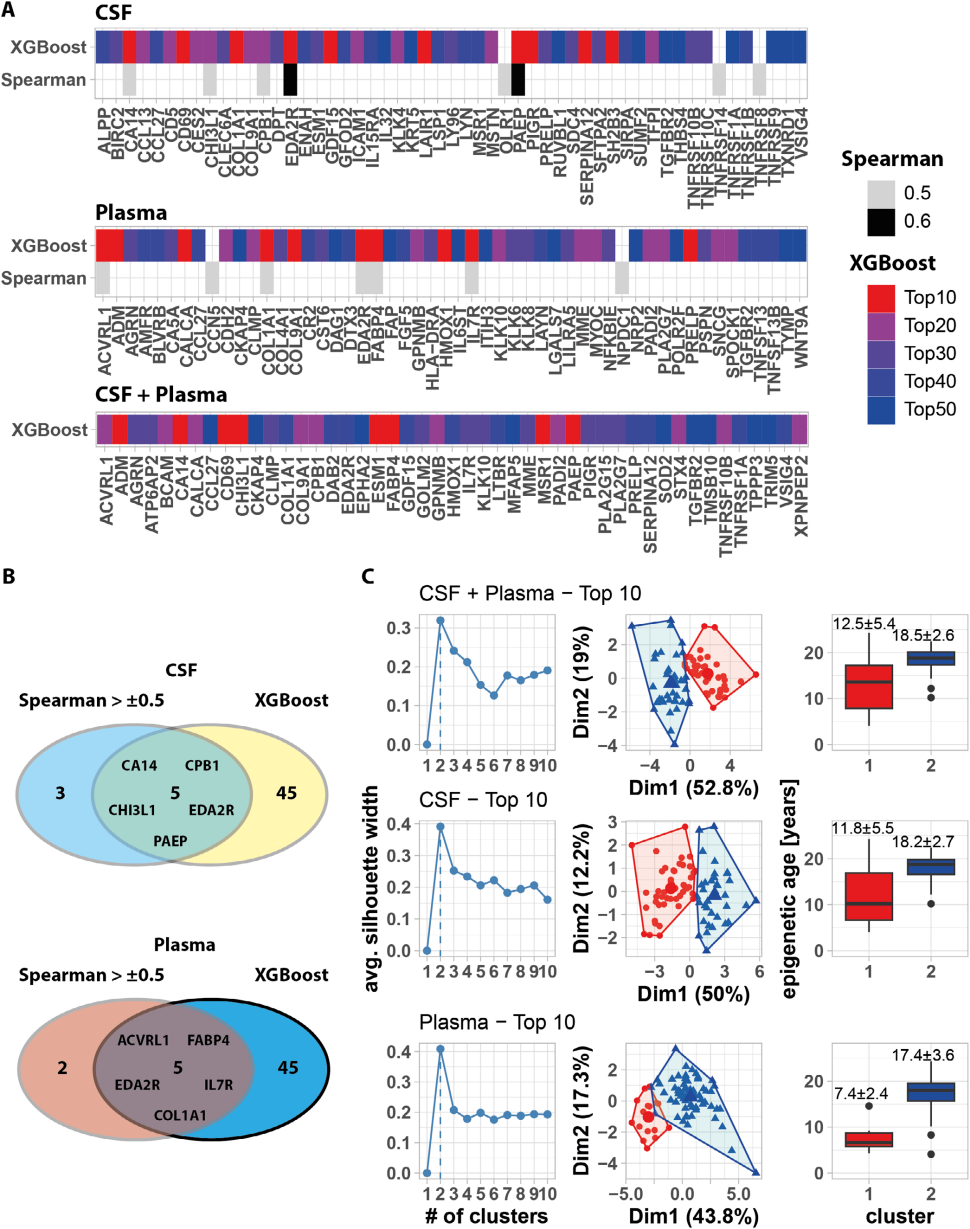
Comparing proteins with age‐correlated levels and XGBoost high gain protein features. (A) A compilation of CSF and/or plasma proteins with a Spearman correlation between NPX value and epigenetic age >±0.5 or the top 10 to top 50 protein features from XGBoost models trained on CSF and plasma, only CSF, or only plasma protein features. (B) The Venn diagrams highlight the overlap between age‐correlating CSF and plasma proteins (Spearman > ±0.5) and the top 50 proteins contributing to XGBoost modeling. (C) Samples from 81 animals were clustered using k‐means using 10 CSF and/or plasma protein features selected based on their high XGBoost gain value. Shown are silhouette plots (left panel) to establish the number of clusters for the k‐means algorithm, a PCA plot of the k‐means result (middle panel) and the epigenetic age of the animals sorted into the respective cluster (right panel).

**FIGURE 6:**
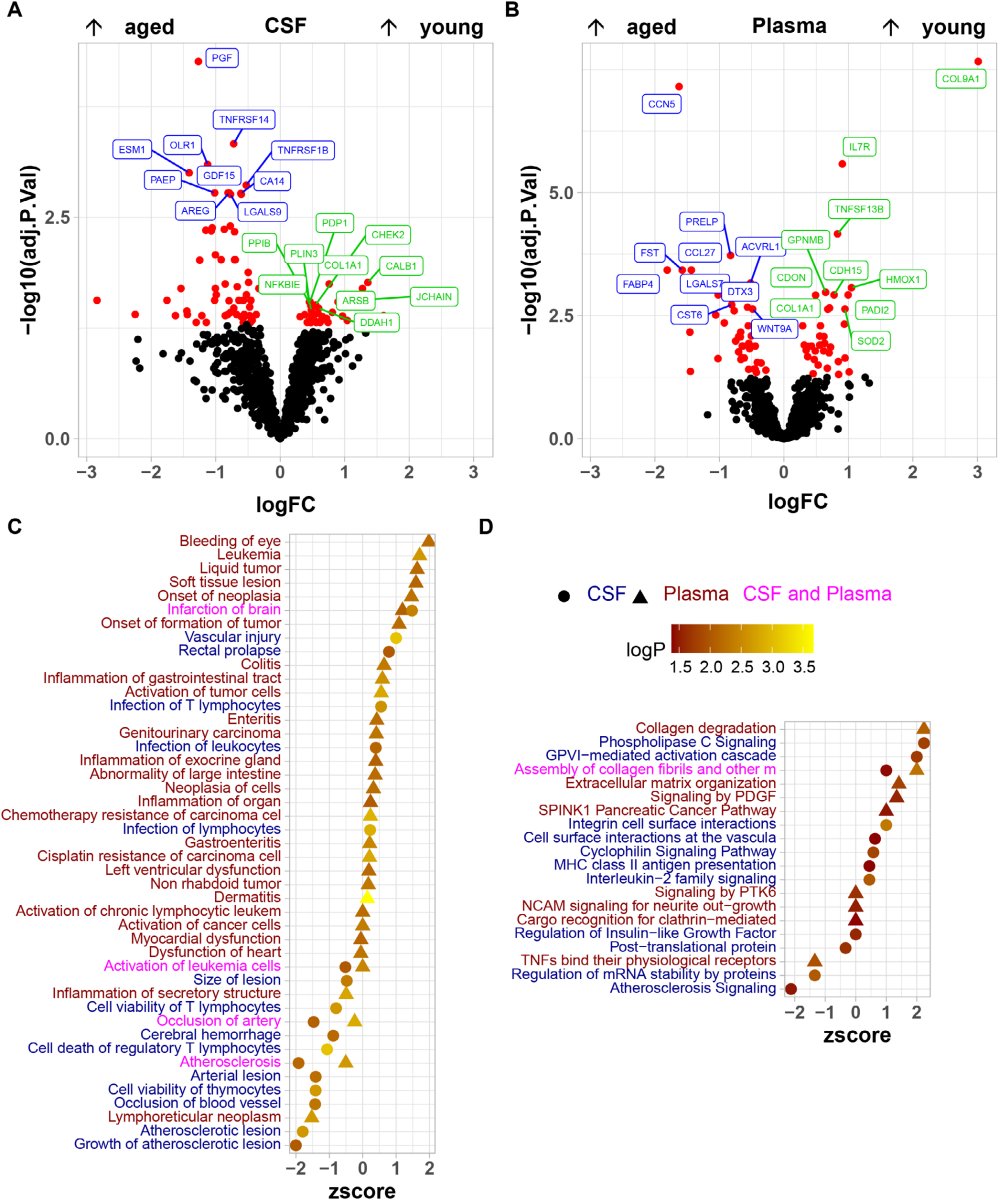
Proteins with age‐related expression pattern are involved in immune‐response pathways. To identify CSF (A) or plasma proteins (B) related to aging, differences in NPX protein values were modeled using limma between animals with an epigenetic age up to 10 years, and animals aged 11 and older. Highlighted in red are proteins with adjusted *p* value < 0.05, labeled are the top 20 (10 up/10 down) CSF and plasma proteins with age‐dependent differences in NPX value and highest *p* value after Benjamini–Hochberg adjustment for multiple testing. Using Ingenuity Pathway Analysis, the connection of age‐regulated CSF and plasma proteins to diseases (C) and regulatory pathways (D) was analyzed. Color‐coding of disease, function, and pathway names highlights involvement of CSF (blue), plasma (brown), or plasma and CSF proteins (magenta).

As XGBoost models outperformed linear model‐based age prediction, we analyzed the overlap between XGBoost high gain features with proteins showing the highest age‐correlated CSF and/or plasma levels based on Spearman coefficients. Interestingly, among the smallest set (*n* = 10) of CSF and plasma protein features used for XGBoost modeling, several correlated with epigenetic age and had a Spearman coefficient > ±0.5 (Figure 5A—CSF: CA14, EDA2R, and PAEP; plasma: ACVRL1, COL1A1, EDA2R, FABP4, and IL7R). Furthermore, out of 15 CSF or plasma protein features correlating with age (Spearman coefficient > ±0.5), 5 did not contribute with high gain to XGBoost models; however, 5 age‐correlating CSF protein features (CA14, CHI3L1, CPB1, EDA2R, and PAEP) and 5 plasma protein features (ACVRL1, COL1A1, EDA2R, FABP4, and IL7R) had predictive value during XGBoost modeling (Figure 5B). The smallest set (*n* = 10) of high gain XGBoost protein features contained some with Spearman correlation < ±0.5 in CSF (CD69, COL1A1, GDF15, LAIR1, PIGR, SERPINA12 and SH2B3) and in plasma (ADM, CALCA, COL9A1, HMOX1, and PRELP). As 10 high gain features were sufficient for generating well‐performing XGBoost models, we used these 10 protein features to perform k‐means clustering (Figure 5C). Using silhouette plots, we first determined 2 as the optimal number of clusters. Plotting the principal component dimensions 1 and 2 illustrated a clear separation of both 2 clusters. Interestingly, cluster separation seemed most prominent when clustering was performed based on the 10 highest gain plasma features. Mean epigenetic age was 12.5 ± 5.4, 11.8 ± 5.5, and 7.4 ± 2.4 years for animals in cluster 1, and 18.5 ± 2.6, 18.2 ± 2.7, and 17.4 ± 3.6 years for animals in cluster 2 when considering the highest gain CSF + plasma, CSF, or plasma XGBoost features, respectively. Based on these clustering results, we categorized animals into 2 age groups (<=10 and > 10) for differential protein level analysis using limma. We found 74 plasma and 93 CSF proteins to have significantly different levels between young (≤ 10 years) and aged (> 10 years) animals (Figure 6A,B). Table S3 lists the most relevant epigenetic age‐related proteins in CSF and plasma identified by ANOVA and XGBoost together with notation of their biological function and any previous associations with age.

Using Ingenuity Pathway Analysis (Kramer et al. 2014) on age‐regulated CSF and plasma proteins, we evaluated whether specific diseases, functions, or molecular pathways were affected by aging. Overall, aging affected 49 disease processes (Figure 6C—CSF: 18; plasma: 31), as well as 21 molecular pathways (Figure 6D—CSF: 12; plasma: 9) with IPA activation *z*‐scores between ±2.2 (Tables S4 and S5).

## 4 Discussion

The present study was successful in demonstrating a highly significant statistical relationship between chronological age of St Kitts African green monkeys and their epigenetic age, as determined by an analysis of discrete methylation sites in DNA extraction from each blood sample. The Mean Absolute Error is 1.2 years (*r* = 0.97) which compares favorably with MAE 1.6 (*r* = 0.95) obtained for rhesus monkey (Horvath et al. 2021), the only other Old World monkey to have a species‐specific epigenetic blood clock. The availability of this bioinformatics tool allows estimation of the epigenetic age of African green monkeys. Previous evidence has demonstrated that epigenetic clocks capture aspects of biological aging and that their rate of change can alter over time in response to relatively short‐term experimental or environmental perturbations (Lu et al. 2020; Stubbs et al. 2017). Unlike chronological age, epigenetic age is derived by bioinformatics and necessarily associated with a degree of error; however, an advantage of using epigenetic age rather than chronological age in the current study is that the proteins associated with epigenetic age should provide insights into biological aging, which is more relevant than chronological age in understanding aging mechanisms and identifying antiaging intervention strategies.

Several studies have examined the relationship between human aging and changes in blood plasma or serum (Argentieri et al. 2024; Coenen et al. 2023; Johnson et al. 2020; Kuo et al. 2024; Sathyan et al. 2020) or CSF (Baird et al. 2012; Zhang et al. 2005) proteomes. While each of these revealed proteins that alter in abundance in association with age, it is notable that there is a lack of consistency among the studies in the specific proteins identified, doubtless due to factors such as the diversity in subject genetics, lifestyle, and environmental exposures, in addition to differences in the proteomic platform employed and the method of quantification. A key advantage to the use of St Kitts origin African green monkeys for proteomic studies is their high level of homozygosity due to the geographic isolation of the population that propagated from only a few founder members originally introduced to the island (Jasinska et al. 2013). Furthermore, the animals in this study would have experienced a more uniform array of environmental factors than subjects recruited for the human studies, with high technical consistency in specimen collection and analysis. There have been very few previous reports on the impact of age on the plasma or CSF proteome in nonhuman primates (Liu et al. 2024; Varma et al. 2024) and none that compared these 2 proteomes in the same subjects across the lifespan of the species.

ANOVA recognized proteins that exhibited a change in abundance across age groups and for which age alone accounted for a high degree of their variability. Notably, several of these age‐related identified proteins in CSF, such as members of the tumor necrosis factor (TNF) receptor superfamily, have critical roles in regulating immune responses, inflammation, and cell survival (Aggarwal 2003; Hunter and Jones 2015). Interestingly, while strong links between aging and levels of EDA2R in blood have been previously noted (Harris et al. 2020; Maggio et al. 2006), the present finding of an association between these proteins in CSF and aging has not been reported before. No proteins were defined by ANOVA as being significantly altered across each age bin in both CSF and plasma together.

An ANOVA is unable to discern patterns of association between factors in a large dataset such as that generated by Olink. We were specifically interested in deriving a small subset of proteins in CSF, plasma, or both fluids that would reliably predict age and to compare the estimated accuracy of such a protein clock with our epigenetic clock. As the study population contained animals of a wide range of ages, statistical grouping of animals into young and aged based on differential protein abundances was not straightforward. Initial PCA and k‐means clustering based on the entirety of measured proteins did not result in the clear separation of animals into two or more age groups. This is likely due to the large number of age‐independent proteins confounding clustering into discernible age groups. Therefore, we focused on sub‐groups of proteins with increasingly stronger associations with age. Using this approach, we identified a Spearman correlation coefficient cut‐off of ±0.4 that only took proteins with a strong age‐dependent component into account for clustering. While this allowed the grouping of animals meaningfully into young (≤ 10 years) and aged (> 10 years), we reasoned that a simple Spearman cut‐off might miss proteins contributing to aging with a less clear level‐age relationship.

An alternative strategy utilizing XGBoost regularizing gradient boosting was better adapted to the Olink dataset, as reviewed by (Salehi et al. 2022). Importantly, and in contrast to other machine learning approaches such as neural networks, XGBoost allows not only extraction of features that contribute to the predictive model but also reports their relative importance. An initial model trained on all proteins in either CSF or plasma predicted epigenetic age with a mean error of 3.7 and 3.0 years, respectively, associated with highly significant linear regressions. Encouraged by this finding, we explored whether improved predictive power could be achieved with a reduced set of proteins for epigenetic age regression. Remarkably, we found that XGBoost models based on 10 highest gain CSF or plasma features predicted epigenetic age with mean errors of ±2.7 and ±2.5 years, respectively, with normal residual distribution. K‐means clustering analysis was then based on these top 10 proteins in CSF or in plasma, which yielded 2 distinct clusters approximately representing ages equal to or less than 10 years and over 10 years of age. This observation has an interesting parallel with a recent human study, which revealed nonlinear patterns in molecular markers of aging, with substantial dysregulation occurring at two major periods occurring at approximately 44 years and 60 years of chronological age (Shen et al. 2024).

Age‐related proteins found in the present study comprised some that had previously been linked with chronological progression and also disclosed several that had not previously been associated with aging in human studies. Those latter proteins could either represent nonhuman primate‐specific aging proteins or those that were obscured by confounding factors in human‐based studies. A comparison of the best blood protein predictors of human chronological age in the large recent study by Argentieri et al. (2024) with the optimized protein predictors of epigenetic age in the current nonhuman primate study (Figure S8) revealed many targets in common, whose relationships to aging mechanisms now deserve further attention. The statistical relationship between the level of individual proteins and epigenetic aging is noteworthy. For example, linear regression analyses revealed that over 40% of the variance in CSF EDA2R levels and CSF CHI3L1 levels was explained by epigenetic aging. In addition, Spearman rank correlation of 0.6 or greater existed for CSF EDA2R and PAEP. Not unexpectedly, the 2 statistical approaches (ANOVA, machine learning algorithms) deployed here revealed some overlap of the most strongly age‐associated proteins, but the machine learning models were able to discern relationships between age‐dependent proteins and derive a protein clock based on as low as 10 protein features that accurately predicted epigenetic age. Consequently, future studies utilizing African green monkeys could quantify alterations in aging trajectory using both the epigenetic clock and the protein clock.

Two general details of the identified CSF proteins linked with aging in the current study deserve further comment. Firstly, some of the proteins in CSF that were associated with aging do not have a well‐defined role in the CNS (e.g., PAEP and REG1A) and this observation warrants further investigation of the possible function of such proteins in brain biochemistry. However, another observation, a tendency for CSF protein levels to increase with age, may at least in part explain the first point noted above. Proteins detected in the CSF are produced either by brain cells, the choroid plexus, or derived from the plasma by leaking through either the blood–brain barrier or the blood‐CSF barrier. It is now appreciated that the blood–brain barrier functions less effectively in aged subjects (Yang et al. 2020), essentially allowing higher molecular weight proteins to cross into the CNS more easily in older compared with younger subjects, with evidence for dysregulation of the blood–brain barrier becoming further exacerbated in certain neurodegenerative disorders (Knox et al. 2022). Secondly, a reduction in CSF turnover occurs during aging, which has a significant concentrating effect on CSF proteins and has been suggested as the major determinant in causing CSF protein concentrations to increase during aging (Chen et al. 2012). Consequently, the presence of a protein in CSF cannot be linked with certainty to its origin in CNS, and a change in concentration of a protein in CSF during aging cannot be assumed to be associated with altered CNS production of the protein. Despite such uncertainties surrounding the reason for detecting certain levels of protein in CSF, inclusion of them in a protein clock as a biomarker of aging is still a valid strategy.

Using a blood epigenetic clock, the current study identified age‐related proteins circulating in body fluids that can be repeatedly and readily obtained, which is an advantage as these types of samples will have most utility in future longitudinal studies of aging in African green monkeys (and other species). While epigenetic clocks based on a specific tissue predict aging in that tissue somewhat better than blood clocks or universal clocks, the ability of blood epigenetic clocks to accurately predict age in different tissues indicates strong conservation of epigenetic aging mechanisms in different tissues (Hannum et al. 2013; Richardson et al. 2025). In addition, the ability of clocks generated in one species to accurately predict age in other species (Jasinska et al. 2022; Lu et al. 2023) supports the relevance to other primate species, including humans, of the presently derived African green monkey blood epigenetic clock and associated proteomic data. Subsets of proteins currently identified in plasma and CSF accurately track epigenetic age in African green monkeys, but it should be noted that recent human studies have modeled plasma proteomic data to detect organ‐specific aging and organ‐specific disease (Goeminne et al. 2025). Thus, there is opportunity for future studies in African green monkeys to tune epigenetic and proteomic models to be more responsive to changes in specific tissues and organs either by constructing tissue‐specific clocks or pan‐ or universal clocks with validation by biomarker evaluation at the tissue level.

Analysis of the biochemical processes that are linked with the age‐associated proteins indicated that pathways activated in older compared to younger animals involves immunological processes as well as pathways related to tissue homeostasis and wound healing. These observations are consistent with a higher need for tissue repair and a general pro‐inflammatory environment in aged animals. Interestingly, such a pro‐inflammatory response was not only seen in plasma, but also in CSF of aged animals. Chronic low‐grade inflammation (“inflammaging”) is a hallmark of aging, is linked to the pathogenesis of many age‐related diseases, and is characterized by chronic activation of the innate immune system (Dugan et al. 2023). The relevance of inflammaging to the progression of aging is supported by studies in long‐lived mice (Arranz et al. 2010) and centenarians (Arai et al. 2015), who exhibit relatively low levels of systemic inflammation. Although a small subset of proteins statistically linked with aging in the present study can together form an impressively accurate protein clock to predict epigenetic and biological aging, it does not quite match the strength of correlation between age and DNA methylation in the African green monkey. However, much more is known of the function of the individual proteins in a protein clock than is understood about the influence that methylation exerts on the separate CpG sites that comprise the DNA clock. This offers the prospect of tailoring a protein clock to a particular physiological function in addition to a version responsive to the more global effects of aging. For example, a subset of CSF or plasma proteins involved in inflammation that have a high statistical link with aging could be combined to form a protein inflammaging clock. In addition, because of the evidence for inflammation being an important component of Alzheimer disease (Heneka et al. 2024), changes in the plasma or CSF proteomic markers could be a valuable approach to tracking progression or amelioration of the pathology in African green monkey models, such as in induced (Wakeman et al. 2022) or age‐related (Corey et al. 2023; Latimer et al. 2019; Varma et al. 2024) models of Alzheimer's disease. Varma et al. (2024) used circumscribed proteomic platforms to explore the relationship between changes in plasma and CSF proteins as a function of aging, sex, cognitive decline, and levels of the putative Alzheimer's disease biomarkers in African green monkeys. By reporting associations between these multiple endpoints, including changes in proteins related to immune‐related inflammation, metabolism, and cellular processes, this study strongly supports the use of African green monkeys in age‐related studies.

Collectively, this study demonstrates that large‐scale plasma and CSF proteomics coupled with machine learning can be leveraged to measure and track aging in nonhuman primates by minimally invasive means. The derivation of accurate CSF and plasma protein clocks for the African green monkey will enable longitudinal evaluation of changes in a small subset of proteins to be a reliable indicator of epigenetic age and biological age in the African green monkey and should also be of relevance to tracking biological age‐related changes in other species of Old World monkeys and humans. The availability of these tools in addition to the blood epigenetic clock for the African green monkey should enable the testing of novel antiaging interventions and their translation to humans to extend healthspan. The blood and CSF clocks constructed here could be further informed by additional analyses of CSF and plasma from animals at the very young and old ranges of the population demographic. Future studies in African green monkeys will further validate the accuracy of protein clocks in predicting epigenetic age and delineate the relationship between these indices of aging in this species with telomere shortening, senescent cell accumulation, and behavioral hallmarks of aging, such as muscle strength, motor coordination, and cognitive performance.

## Author Contributions

J.D.E., D.R.W., and M.S.L. conceived the study; J.D.E. and D.R.W. collected the data; A.N., J.R., J.G., M.M., and L.D. analyzed the data; J.D.E. and A.N. wrote the manuscript; J.R., M.M., M.S.L., D.R.W., B.D., and C.A. revised the manuscript.

## Conflicts of Interest

The authors declare no conflicts of interest.

## Supporting information


Figures S1–S8.

Tables S1–S5.

## Data Availability

All data that support the findings of this study are available from the authors upon request.
