# Supplementary material for: Proteomic Signatures of Epigenetic Age in African Green Monkey Cerebrospinal Fluid and Plasma
Source: Aging Cell. 2025 Jul 29;24(10):e70168. doi: 10.1111/acel.70168 (PMC12507418; doi:10.1111/acel.70168)
Supplement: Supplementary file 1 — Figures S1–S8. [file ACEL-24-e70168-s002.docx]

**Supporting Figures**

**
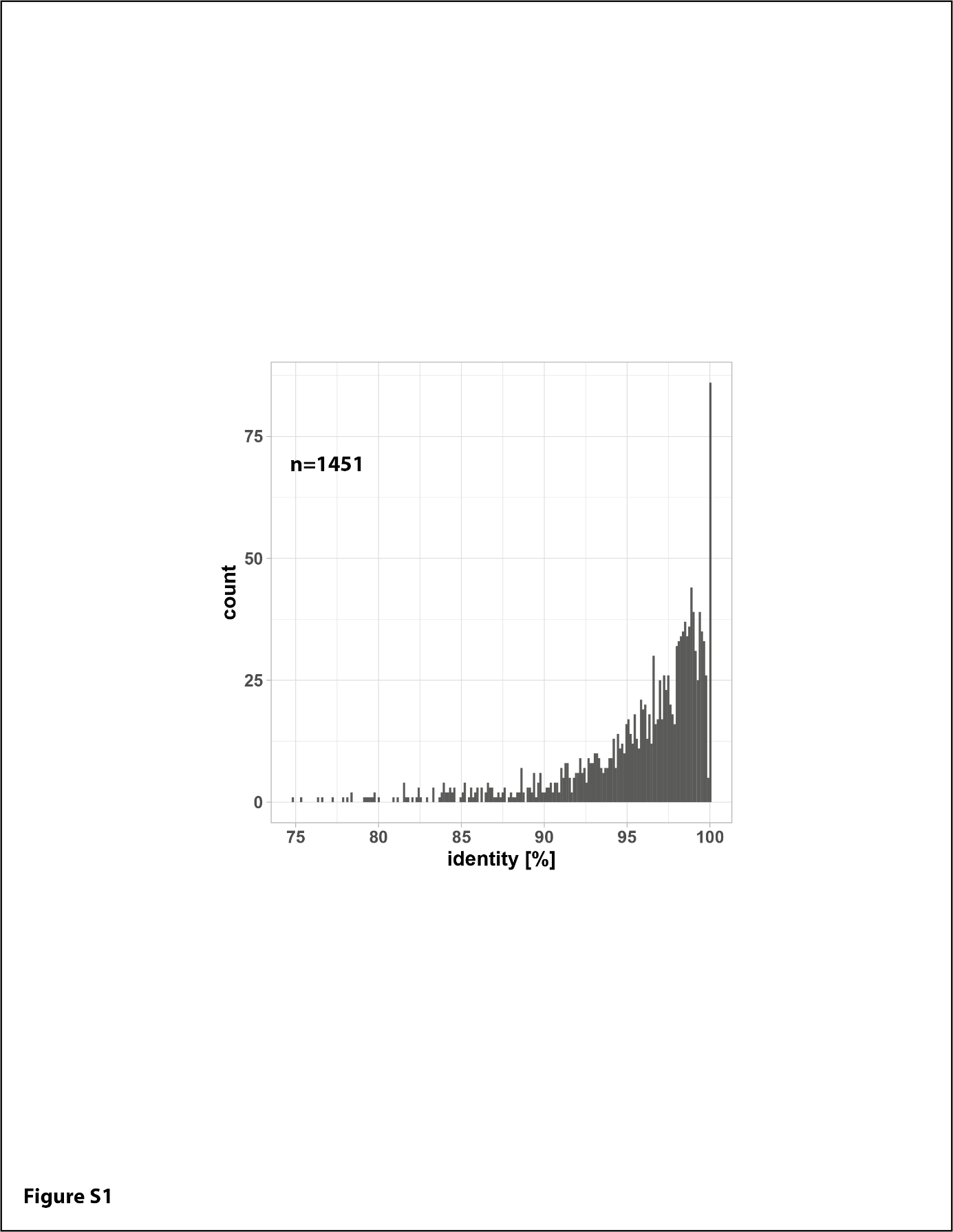
**

**Figure S1** *Conservation of Olink-detected proteins between African Green monkey and human.* Sequences of human proteins analyzed in this study using Olink were aligned using BLAST to all annotated *Chlorocebus sabeus* proteins in the Uniprot and NCBI protein databases. Shown are % identity reported by BLAST. Out of 1456 human protein sequences retrieved, 1451 could be matched to *Chlorocebus sabeus* orthologs. Due to low similarity, five proteins were excluded following manual confirmation.


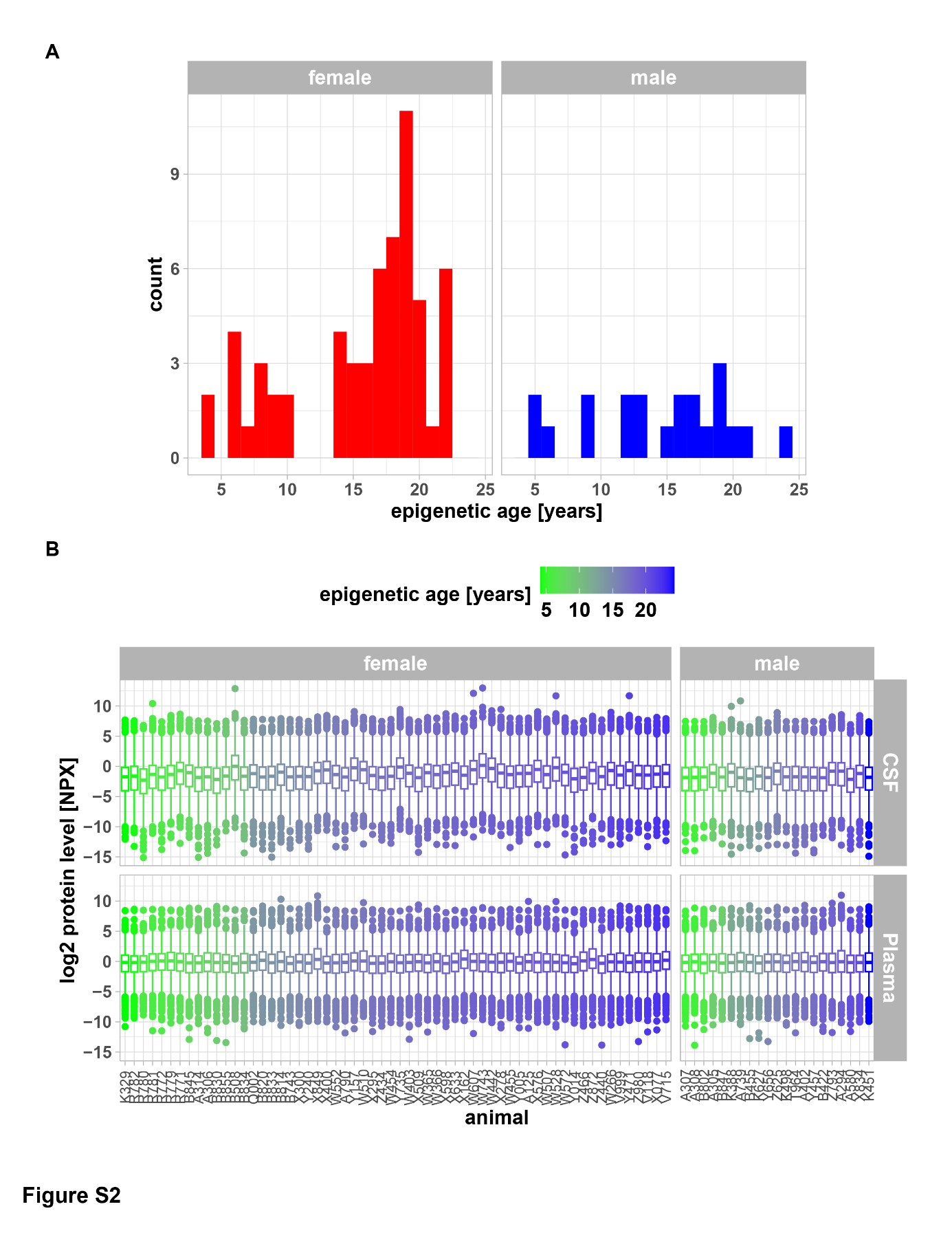


**Figure S2** *Distribution of study animal age and CSF or plasma protein levels* (A) In the present study, 81 *Chlorocebus aethiops* Old World monkeys were included. Shown is the epigenetic age distribution of female and male study animals. (B) Using Olink technology, levels of 1472 CSF and plasma proteins were determined in 81 animals. The boxplots represent normalized log2 protein levels expressed in Olink-specific NPX values.

**
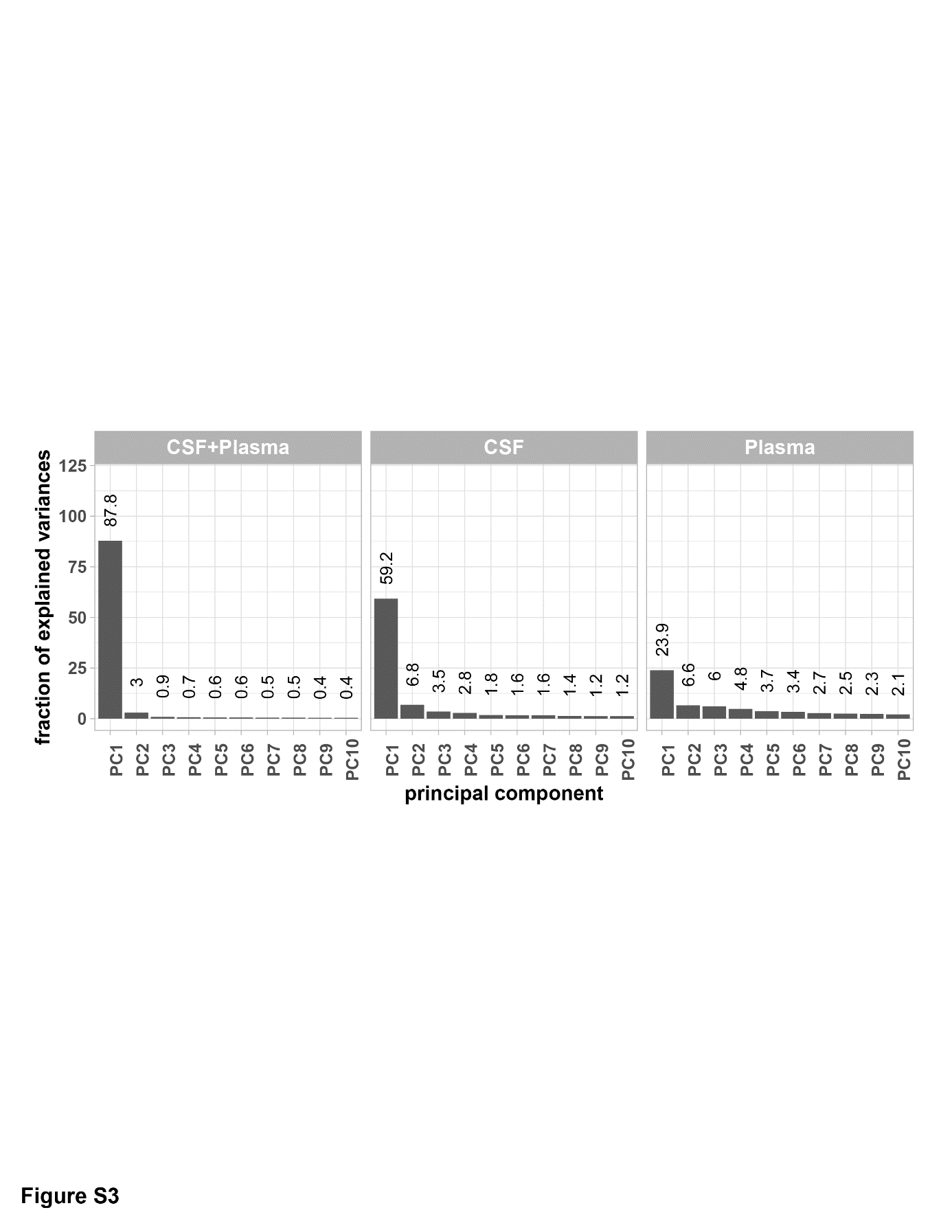
**

**Figure S3** *Explained variance by principal component.* Scree plots for PCAs detailing variance explained by principal component. Compare to Figure 2A-C.


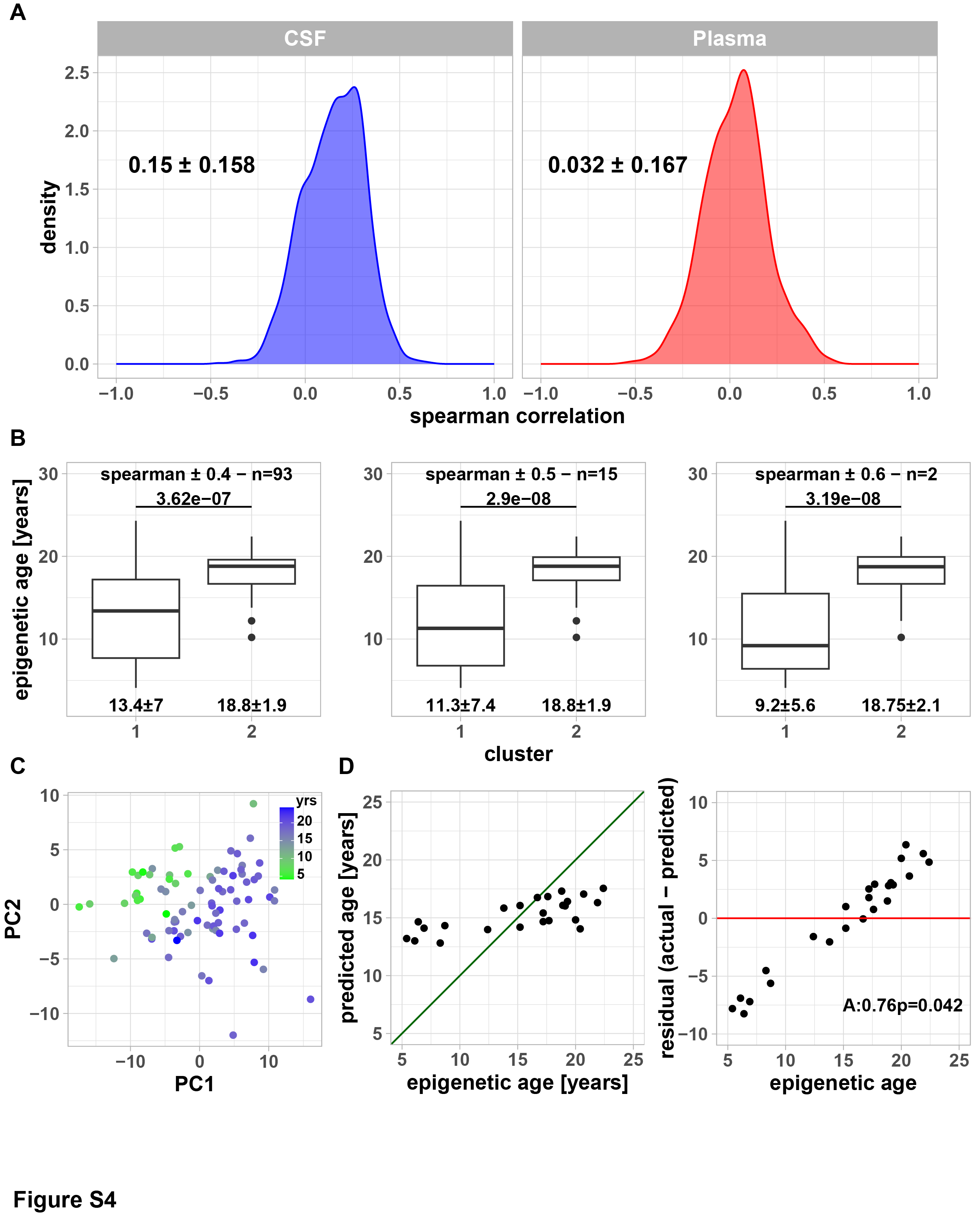


**Figure S4:** *Proteins with age-correlated levels in CSF and plasma.* (A) Using Spearman correlation, the relation between levels of plasma or CSF proteins and their epigenetic age was analyzed. Annotated are mean Spearman correlation ± standard deviation. (B) K-means clustering was performed on NPX values of CSF and plasma proteins with a Spearman correlation coefficient between epigenetic age and NPX protein levels > ±0.4 to ±0.6. Annotated are numbers of protein features (n) and mean epigenetic age in years ± standard deviation of the two identified clusters. (C) PCA of samples with data restricted to 93 NPX protein with Spearman correlation between epigenetic age and NPX protein levels > ± 0.4. (D) Linear regression for 38 plasma and 55 CSF proteins with a Spearman correlation > ±0.4 between epigenetic age and NPX protein level was performed on a random 70:30 split of the input data into a train and a test dataset. The epigenetic age of animals in the test dataset was predicted by calculating and averaging the results of these 93 linear models. Shown is the actual versus the calculated epigenetic age (upper panel) and the residual plot (lower panel) with the expected result marked in green or red, respectively.

**
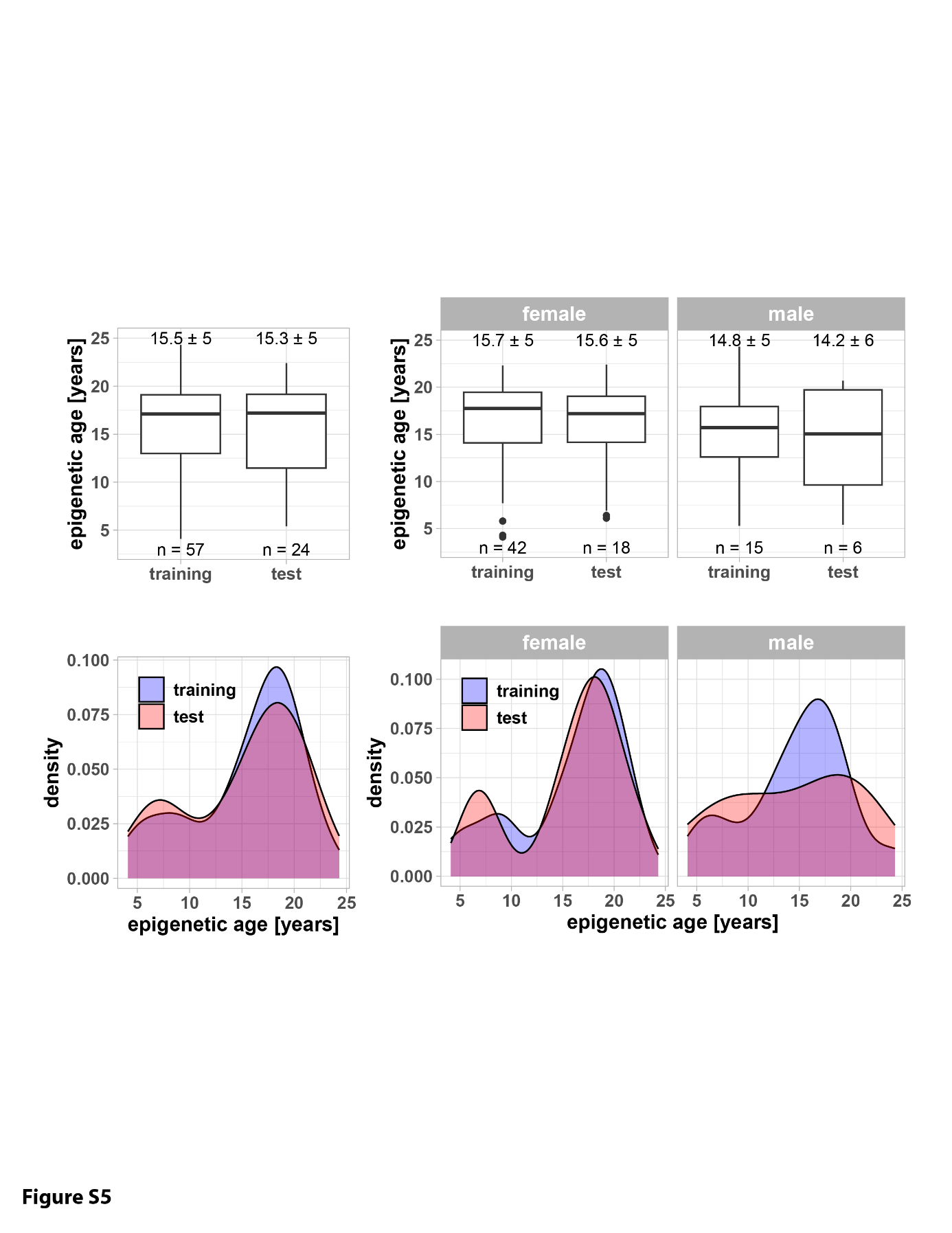
**

**Figure S5:** *Splitting study population into a training and test data set.* 81 samples were split 70:30 into a training (n=57) and holdout (n=24) dataset with similar mean age and age distribution between train and test dataset. The training set was used for model generation, while the test data set served for model validation.


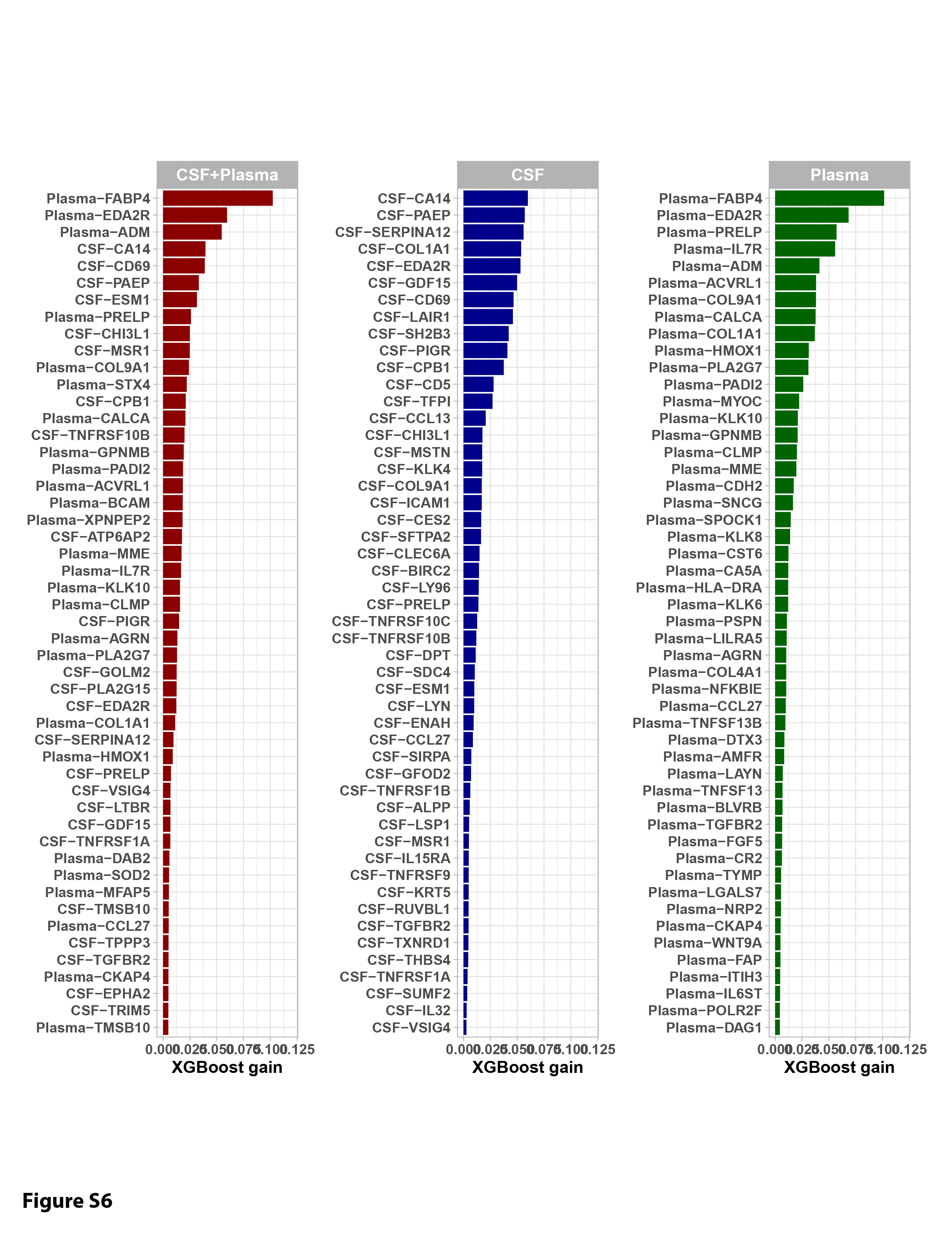


**Figure S6:** *Selection of CSF and plasma proteins contributing with high gain to XGBoost models.* Gain values were extracted from XGBoost models trained on CSF and/or plasma protein expression levels. Shown are 50 protein features with the highest gain for the top performing model trained on CSF+Plasma (red), CSF (blue), or Plasma (green) features, respectively.

**
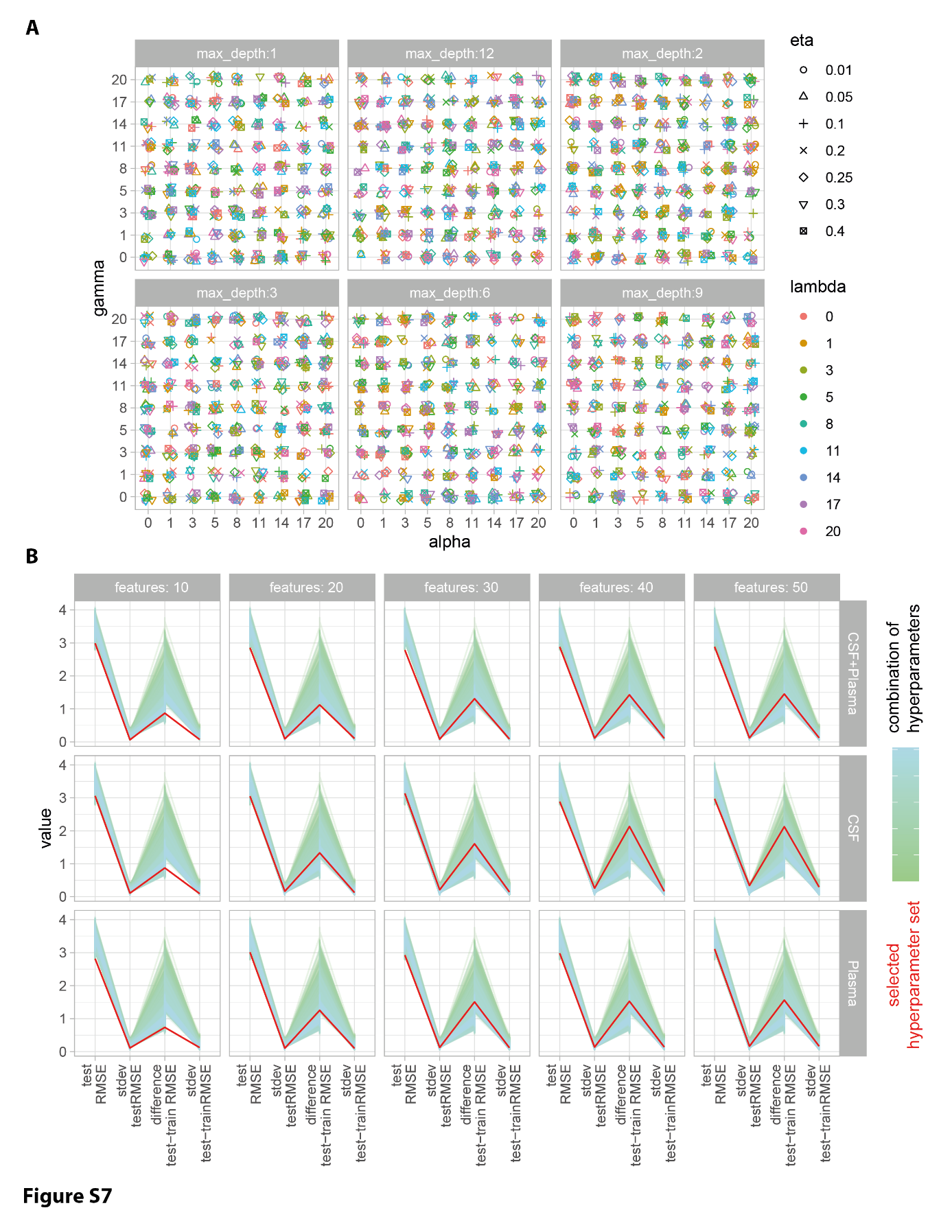
**

**Figure S7:** *Restricting XGBoost feature space.* (A) For tuning of XGBoost hyperparameters, learning rate (eta), maximum decision tree depth (max_depth), L1 (alpha) L2 (lambda) weight regularization terms, and minimum loss reduction for split gamma were varied as indicated to generate a 30619 row parameter matrix. The plot shows 3422 hyperparameter combinations randomly selected and used for XGBoost model tuning. (B) A parallel coordinate plot showing the result of XGBoost model tuning based on 3422 hyperparameter sets evaluated 20-times with different random start seeds and with 10 to 50 CSF or plasma protein features (NPX values of CSF or plasma samples from 57 animals of the train dataset – Figure S5). Shown are root mean square error (RMSE) of test validation reported by XGBoost, difference between test and train RMSE as well as their respective standard deviation. The best performing hyperparameter set for use in model training and prediction of the test/holdout dataset (marked red) was selected based on minimal Euclidean distance to zero of these four tuning results.

**
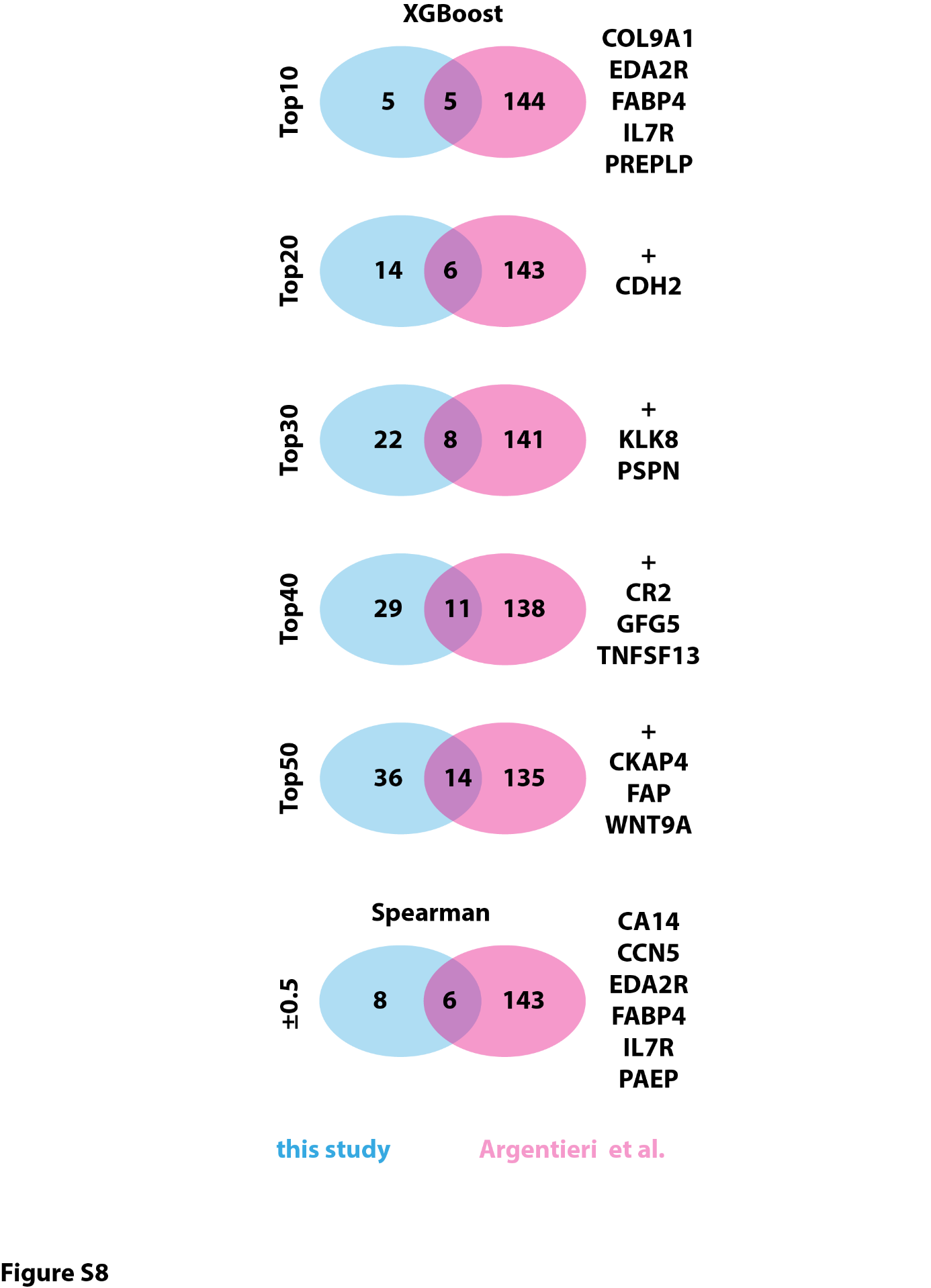
**

**Figure S8:** *Comparing human to AGM age-predicting protein sets.* The overlap between age-predicting proteins identified by Argentieri et al. and proteins identified in this study was analyzed. Out of 204 (Argentieri) proteins identified as age-predicting, protein levels of 149 proteins were determined in this study. The Venn diagrams show overlap between protein sets predictive in XGBoost modeling (Top10 to Top50 proteins) or age-correlating proteins (Spearman) with these external protein sets. Proteins shared between two compared sets are annotated*.*
