# Supplementary material for: Proteomic Signatures of Epigenetic Age in African Green Monkey Cerebrospinal Fluid and Plasma
Source: Aging Cell. 2025 Jul 29;24(10):e70168. doi: 10.1111/acel.70168 (PMC12507418; doi:10.1111/acel.70168)
Supplement: Supplementary file 2 — Tables S1–S5. [file ACEL-24-e70168-s001.docx]

**Supporting Tables**

**Table S1: Epigenetic age-related CSF and plasma proteins identified by ANOVA**

**Table S1A: CSF proteins with significant changes in abundance in the same direction between each age group**

| **Protein** | **Direction** | **Age group contrast, p value*** | | | **Regression Analysis** | |
| --- | --- | --- | --- | --- | --- | --- |
|  |  | **Y**→**M** | **M**→**A** | **Y**→**A** | **p value** | **R2 (%)** |
| EDA2R | ↑ | 4 x 10-6 | 3 x 10-2 | 3 x 10-9 | 3 x 10-11 | 0.42 |
| PAEP | ↑ | 2 x 10-5 | 2 x 10-3 | 4 x 10-10 | 7 x 10-11 | 0.41 |

**Table S1B: The 10 CSF and 10 plasma proteins with the most significant contrasts between 2 of the 3 age groups**

|  | **Protein** | **Direction** | **p value*** | | | **Regression Analysis** | |
| --- | --- | --- | --- | --- | --- | --- | --- |
| **Source** |  |  | **Y**→**M** | **M**→**A** | **Y**→**A** | **p value** | **R^2^ (%)** |
| CSF | CHI3L1 | ↑ | 2 x 10-10 | 0.09 (NS) | < 1 x 10-15 | 2 x 10-14 | 0.52 |
| CSF | TNFRSF14 | ↑ | 2 x 10-6 | 0.21 (NS) | 4 x 10-8 | 8 x 10-10 | 0.37 |
| CSF | REG1A | ↑ | 2 x 10-6 | 0.41 (NS) | 3 x 10-7 | 9 x 10-10 | 0.37 |
| CSF | CPB1 | ↑ | 4 x 10-6 | 0.14 (NS) | 5 x 10-8 | 1 x 10-9 | 0.36 |
| CSF | OLR1 | ↑ | 8 x 10-6 | 0.25 (NS) | 3 x 10-7 | 3 x 10-10 | 0.38 |
| CSF | LAIR1 | ↑ | 5 x 10-6 | 0.99 (NS) | 7 x 10-7 | 3 x 10-7 | 0.28 |
| CSF | CA14 | ↑ | 8 x 10-5 | 0.10 (NS) | 5 x 10-7 | 6 x 10-9 | 0.34 |
| CSF | TNFRSF1B | ↑ | 1 x 10-4 | 0.35 (NS) | 8 X 10-6 | 5 x 10-7 | 0.27 |
| CSF | ADA2 | ↑ | 6 x 10-5 | 0.65 (NS) | 2 X 10-5 | 6 x 10-6 | 0.22 |
| CSF | GDF15 | ↑ | 7 x 10-5 | 0.78 (NS) | 6 x 10-5 | 1 x 10-6 | 0.25 |
| Plasma | COL9A1 | ↓ | < 1 x 10-15 | 0.80 (NS) | < 1 x 10-15 | 7 x 10-14 | 0.53 |
| Plasma | CCN5 | ↑ | 6 x 10-8 | 0.97 (NS) | 4 x 10-8 | 2 x 10-8 | 0.34 |
| Plasma | IL7R | ↓ | 5 x 10-7 | 0.87 (NS) | 6 x 10-8 | 2 x 10-8 | 0.34 |
| Plasma | PRELP | ↑ | 7 x 10-7 | 0.88 (NS) | 2 x 10-7 | 6 x 10-8 | 0.32 |
| Plasma | FABP4 | ↑ | 2 x 10-6 | 0.92 (NS) | 6 x 10-7 | 4 x 10-8 | 0.33 |
| Plasma | COL1A1 | ↓ | 4 x 10-6 | 0.79 (NS) | 5 x 10-7 | 2 x 10-9 | 0.38 |
| Plasma | TNFSF13B | ↓ | 3 x 10-6 | 0.98 (NS) | 9 x 10-7 | 2 x 10-6 | 0.26 |
| Plasma | GPNMB | ↓ | 4 x 10-5 | 0.51 (NS) | 8 x 10-7 | 4 x 10-6 | 0.24 |
| Plasma | CST6 | ↑ | 8 x 10 -6 | 0.93 (NS) | 3 x 10-6 | 1 x 10-6 | 0.26 |
| Plasma | ACP5 | ↓ | 3 x 10-5 | 0.99 (NS) | 2 x 10-5 | 1 x 10-6 | 0.27 |

**p values for pairwise contrast of age groups: Y, young; M, middle-aged; A, aged. An up or down arrow in direction indicates and increase or decrease respectively in protein abundance with advancing age.*

**Table S2: Age-correlating proteins with Spearman Rank Correlation greater ± 0.4**

| **Protein** | **Sample** | **Spearman Rank Correlation** |
| --- | --- | --- |
| ACVRL1 | Plasma | 0.54 |
| ADAM22 | Plasma | 0.4 |
| ADM | Plasma | 0.49 |
| AGRN | Plasma | 0.45 |
| CALCA | Plasma | 0.45 |
| CCL27 | Plasma | 0.41 |
| CCN5 | Plasma | 0.51 |
| CD70 | Plasma | 0.41 |
| CDH15 | Plasma | -0.4 |
| CDON | Plasma | -0.42 |
| CELA3A | Plasma | 0.41 |
| CLMP | Plasma | 0.46 |
| COL1A1 | Plasma | -0.51 |
| COL9A1 | Plasma | -0.49 |
| CR2 | Plasma | -0.47 |
| CRIP2 | Plasma | 0.41 |
| CST6 | Plasma | 0.44 |
| CXCL14 | Plasma | 0.42 |
| EDA2R | Plasma | 0.56 |
| ERBB4 | Plasma | 0.42 |
| FABP4 | Plasma | 0.51 |
| FGFR2 | Plasma | 0.44 |
| HMOX1 | Plasma | -0.42 |
| IL7R | Plasma | -0.55 |
| KLK6 | Plasma | 0.45 |
| MDK | Plasma | 0.43 |
| MSTN | Plasma | -0.43 |
| NPDC1 | Plasma | 0.51 |
| PAMR1 | Plasma | -0.43 |
| PRELP | Plasma | 0.47 |
| PRSS2 | Plasma | 0.43 |
| PSPN | Plasma | 0.42 |
| TGFBR2 | Plasma | 0.41 |
| TNFRSF19 | Plasma | 0.48 |
| TNFSF13 | Plasma | 0.44 |
| TNR | Plasma | 0.42 |
| WFDC2 | Plasma | 0.4 |
| WNT9A | Plasma | 0.46 |
| AGRN | CSF | 0.42 |
| AREG | CSF | 0.43 |
| CA14 | CSF | 0.57 |
| CCL2 | CSF | 0.47 |
| CCL4 | CSF | 0.43 |
| CD69 | CSF | 0.48 |
| CD74 | CSF | 0.42 |
| CELA3A | CSF | 0.42 |
| CHI3L1 | CSF | 0.6 |
| CNDP1 | CSF | 0.44 |
| CPA1 | CSF | 0.44 |
| CPB1 | CSF | 0.52 |
| CST3 | CSF | 0.43 |
| CST6 | CSF | 0.48 |
| CTSS | CSF | 0.42 |
| DDR1 | CSF | 0.41 |
| EDA2R | CSF | 0.62 |
| ERBB3 | CSF | 0.45 |
| ESM1 | CSF | 0.48 |
| F3 | CSF | 0.42 |
| FGFR2 | CSF | 0.45 |
| GDF15 | CSF | 0.46 |
| HAVCR2 | CSF | 0.48 |
| IL18 | CSF | 0.4 |
| IL6R | CSF | 0.46 |
| KLK6 | CSF | -0.45 |
| LAIR1 | CSF | 0.46 |
| LGALS9 | CSF | 0.44 |
| LTBP2 | CSF | 0.43 |
| MMP10 | CSF | 0.42 |
| MSR1 | CSF | 0.48 |
| NBL1 | CSF | 0.46 |
| NCAM2 | CSF | 0.43 |
| NEFL | CSF | 0.43 |
| OLR1 | CSF | 0.55 |
| OXT | CSF | 0.47 |
| PAEP | CSF | 0.66 |
| PDGFA | CSF | 0.43 |
| PGF | CSF | 0.47 |
| PLAUR | CSF | 0.44 |
| REG1A | CSF | 0.47 |
| REG1B | CSF | 0.48 |
| TEK | CSF | 0.42 |
| TFPI | CSF | 0.5 |
| TFPI2 | CSF | 0.4 |
| TNFRSF10B | CSF | 0.48 |
| TNFRSF12A | CSF | 0.42 |
| TNFRSF14 | CSF | 0.55 |
| TNFRSF19 | CSF | 0.43 |
| TNFRSF1A | CSF | 0.47 |
| TNFRSF1B | CSF | 0.48 |
| TNFRSF21 | CSF | 0.42 |
| TNFRSF8 | CSF | 0.54 |
| VCAM1 | CSF | 0.45 |
| VSIG4 | CSF | 0.45 |

**Table S3: Protein biomarkers strongly associated with aging**

| **Protein** | **Human**  **Uniprot ID** | **AGM match** | **% Identity** | **CSF** | **Plasma** | **ANOVA** | **XGBoost** | **Function** | **Aging change** |
| --- | --- | --- | --- | --- | --- | --- | --- | --- | --- |
| CA14 | Q9ULX7 | A0A0D9RZT1 | 96.4 | + | - | + | + | [1] |  |
| CD69 | Q07108 | XP_037851043.1 | 92.7 | + | - | - | + | [2] | [3] |
| CHI3L1 | P36222 | A0A0D9RPC7 | 96.9 | + | - | + | - | [4] | [5] |
| COL1A1 | P02452 | A0A0D9QYW4 | 99.4 | + | + | + | + | [6] |  |
| CPB1 | P15086 | A0A0D9RG86 | 93.9 | + | - | + | - | [7] |  |
| EDA2R | Q9HAV5 | XP_037850604.1 | 92.9 | + | + | - | + | [8] | [9] |
| GDF15 | Q99988 | XP_037848366.1 | 91.9 | + | - | - | + | [10] | [11] |
| LAIR1 | Q6GTX8 | A0A0D9S5M3 | 81.5 | + | - | + | + | [12] |  |
| OLR1 | P78380 | A0A0D9R9R0 | 93.8 | + | - | + | - | [13] | [14] |
| PAEP | P09466 | A0A0D9RSX5 | 83.3 | + | - | + | + | [15] |  |
| PIGR | P01833 | A0A0D9RR96 | 89.1 | + | - | - | + | [16] |  |
| REG1A | P05451 | A0A0D9RTK8 | 94.6 | + | - | + | - | [17] |  |
| SERPINA12 | Q8IW75 | XP_007985898.2 | 90.3 | + | - | - | + | [18] |  |
| SH2B3 | Q9UQQ2 | XP_008002918.1 | 96.1 | + | - |  | + | [19] |  |
| TNFRSF14 | Q92956 | XP_007979094.2 | 83.8 | + | - | + | - | [20] | [21] |
| TNFRSF1B | P20333 | A0A0D9S8K8 | 95.7 | + | - | + | - | [22] | [23] |
| ACP5 | P13686 | A0A0D9R321 | 97.5 | - | + | + | - | [24] | [25] |
| ACVRL1 | P37023 | A0A0D9R0K0 | 98.6 | - | + | - | + | [26] | [27] |
| ADM | P35318 | A0A0D9QX00 | 95.7 | - | + | - | + | [28] | [29] |
| CALCA | P01258 | A0A0D9QXH0 | 93.6 | - | + | - | + | [30] |  |
| CCN5 | O76076 | A0A0D9RNZ1 | 96.4 | - | + | + |  | [31] |  |
| COL9A1 | P20849 | XP_008011772.2 | 97.6 | - | + | + | + | [32] |  |
| CST6 | Q15828 | A0A0D9R6X2 | 96.6 | - | + | + | - | [33] |  |
| FABP4 | Q01469 | A0A0D9RLE5 | 98.5 | - | + | + | + | [34] |  |
| GPNMB | Q14956 | XP_007980080.2 | 94.1 | - | + | + | - | [35] | [36] |
| HMOX1 | P09601 | A0A0D9R769 | 97.2 | - | + | - | + | [37] | [38] |
| IL7R | P16871 | XP_007959554.2 | 96.7 | - | + | + | + | [39] | [40] |
| PRELP | P51888 | A0A0D9RPE4 | 98.4 | - | + | + | + | [41] |  |
| TNFSF13B | Q9Y275 | A0A0D9RY15 | 96.5 | - | + | + | - | [42] | [43] |

Listed are proteins with significant differences between young, middle-aged, and aged animals (marked with ANOVA) and biomarkers identified using XGBoost modelling as most significant (marked with XGBoost). % Identity refers to protein sequence conservation between human and corresponding AGM protein. *Abbreviations for proteins as designated by Olink; many proteins have alternate abbreviations which can be sourced at [44].

**Table S4: Ingenuity Pathway Analysis pathways significantly activated or suppressed based on CSF proteins**

| Pathway | logP | ratio | zscore | molecules |
| --- | --- | --- | --- | --- |
| Integrin cell surface interactions | 2.34 | 0.429 | 1 | AGRN, COL1A1, COL4A1, F11R, ITGA11, ITGAV, JAM2, SPP1, VCAM1 |
| Regulation of mRNA stability by proteins that bind AU-rich elements | 2.02 | 0.556 | -1.342 | EIF4G1, HSPA1A, HSPB1, PSME2, TNFSF13 |
| Interleukin-2 family signaling | 2.02 | 0.556 | 0.447 | HAVCR2, IL2, LGALS9, PTPN6, STAT5B |
| Cyclophilin Signaling Pathway | 1.82 | 0.324 | 0.577 | APP, CCL2, IL2, IL6R, NFATC3, NFKBIE, OLR1, PPIB, PQBP1, SPP1, STAT5B, VCAM1 |
| Phospholipase C Signaling | 1.77 | 0.36 | 2.236 | ARHGEF12, ITGA11, ITGAV, JCHAIN, LAT, LYN, NFATC3, PEBP1, TGM2 |
| GPVI-mediated activation cascade | 1.73 | 0.571 | 2 | COL1A1, LAT, LYN, PTPN6 |
| Post-translational protein phosphorylation | 1.65 | 0.346 | -0.333 | APP, CHGB, CHRDL1, CSF1, FSTL3, IGFBP4, IGFBP7, LGALS1, SPP1 |
| Regulation of Insulin-like Growth Factor (IGF) transport and uptake by IGFBPs | 1.57 | 0.323 | 0 | APP, CHGB, CHRDL1, CSF1, FSTL3, IGFBP4, IGFBP7, LGALS1, PAPPA, SPP1 |
| Atherosclerosis Signaling | 1.47 | 0.312 | -2.121 | CCL2, CD40, COL1A1, CSF1, MSR1, SELPLG, TNFRSF12A, TNFRSF14, TNFSF12, VCAM1 |
| Assembly of collagen fibrils and other multimeric structures | 1.41 | 0.417 | 1 | COL1A1, COL4A1, CTSL, CTSS, CTSV |
| MHC class II antigen presentation | 1.39 | 0.375 | 0.447 | CTSL, CTSO, CTSS, CTSV, LAG3, LGMN |
| Cell surface interactions at the vascular wall | 1.38 | 0.303 | 0.632 | COL1A1, F11R, ITGAV, JAM2, JCHAIN, LYN, OLR1, PTPN6, SELPLG, TNFRSF10B |

**Table S5: Ingenuity Pathway Analysis pathways activated or suppressed based on plasma proteins**

| Pathway | logP | ratio | zscore | molecules |
| --- | --- | --- | --- | --- |
| Assembly of collagen fibrils and other multimeric structures | 2.26 | 0.417 | 2 | COL1A1, COL4A1, COL9A1, CTSV, MMP13 |
| Collagen degradation | 2.09 | 0.385 | 2.236 | COL1A1, COL4A1, COL9A1, CTSD, MMP13 |
| TNFs bind their physiological receptors | 1.94 | 0.357 | -1.342 | CD70, EDA2R, TNFRSF13B, TNFSF13, TNFSF13B |
| Extracellular matrix organization | 1.81 | 0.25 | 1.414 | AGRN, COL1A1, COL4A1, COL9A1, DAG1, ITGA5, NID2, TNC |
| Signaling by PTK6 | 1.65 | 0.364 | 0 | ERBB2, EREG, GPNMB, PXN |
| Signaling by PDGF | 1.56 | 0.294 | 1.342 | COL4A1, COL9A1, PLAT, THBS2, THBS4 |
| NCAM signaling for neurite out-growth | 1.51 | 0.333 | 0 | AGRN, COL4A1, COL9A1, PSPN |
| SPINK1 Pancreatic Cancer Pathway | 1.46 | 0.278 | 1 | CPM, KLK10, KLK13, KLK6, TGFBR2 |
| Cargo recognition for clathrin-mediated endocytosis | 1.39 | 0.308 | 0 | DAB2, EREG, IGF2R, IL7R |

**References for Supplemental Tables**

1. Parkkila S, Parkkila AK, Rajaniemi H, Shah GN, Grubb JH, Waheed A, Sly WS. (2001). Expression of membrane-associated carbonic anhydrase XIV on neurons and axons in mouse and human brain. Proc Natl Acad Sci U S A 98: 1918-23. PubMed PMID: 11172051.

2. Jimenez-Fernandez M, de la Fuente H, Martin P, Cibrian D, Sanchez-Madrid F. (2023). Unraveling CD69 signaling pathways, ligands and laterally associated molecules. EXCLI J 22: 334-51. PubMed PMID: 37223078.

3. Kusdra L, Rempel H, Yaffe K, Pulliam L. (2000). Elevation of CD69+ monocyte/macrophages in patients with Alzheimer's disease. Immunobiology 202: 26-33. PubMed PMID: 10879686.

4. Zhao T, Su Z, Li Y, Zhang X, You Q. (2020). Chitinase-3 like-protein-1 function and its role in diseases. Signal Transduct Target Ther 5: 201. PubMed PMID: 32929074.

5. Connolly K, Lehoux M, O'Rourke R, Assetta B, Erdemir GA, Elias JA, Lee CG, Huang YA. (2023). Potential role of chitinase-3-like protein 1 (CHI3L1/YKL-40) in neurodegeneration and Alzheimer's disease. Alzheimers Dement 19: 9-24. PubMed PMID: 35234337.

6. Li X, Sun X, Kan C, Chen B, Qu N, Hou N, Liu Y, Han F. (2022). COL1A1: A novel oncogenic gene and therapeutic target in malignancies. Pathol Res Pract 236: 154013. PubMed PMID: 35816922.

7. Yu SL, Han S, Kim HR, Park JW, Jin DI, Kang J. (2017). Phosphorylation of carboxypeptidase B1 protein regulates beta-cell proliferation. Int J Mol Med 40: 1397-404. PubMed PMID: 28949379.

8. Cai Z, Deng X, Jia J, Wang D, Yuan G. (2021). Ectodysplasin A/Ectodysplasin A Receptor System and Their Roles in Multiple Diseases. Front Physiol 12: 788411. PubMed PMID: 34938205.

9. Harris SE, Cox SR, Bell S, Marioni RE, Prins BP, Pattie A, Corley J, Munoz Maniega S, Valdes Hernandez M, Morris Z, John S, Bronson PG, Tucker-Drob EM, Starr JM, Bastin ME, Wardlaw JM, Butterworth AS, Deary IJ. (2020). Neurology-related protein biomarkers are associated with cognitive ability and brain volume in older age. Nat Commun 11: 800. PubMed PMID: 32041957.

10. Sigvardsen CM, Richter MM, Engelbeen S, Kleinert M, Richter EA. (2024). GDF15 is still a mystery hormone. Trends Endocrinol Metab: PubMed PMID: 39472228.

11. Conte M, Giuliani C, Chiariello A, Iannuzzi V, Franceschi C, Salvioli S. (2022). GDF15, an emerging key player in human aging. Ageing Res Rev 75: 101569. PubMed PMID: 35051643.

12. Van Laethem F, Donaty L, Tchernonog E, Lacheretz-Szablewski V, Russello J, Buthiau D, Almeras M, Moreaux J, Bret C. (2022). LAIR1, an ITIM-Containing Receptor Involved in Immune Disorders and in Hematological Neoplasms. Int J Mol Sci 23: PubMed PMID: 36555775.

13. Sanchez-Leon ME, Loaeza-Reyes KJ, Matias-Cervantes CA, Mayoral-Andrade G, Perez-Campos EL, Perez-Campos-Mayoral L, Hernandez-Huerta MT, Zenteno E, Perez-Cervera Y, Pina-Canseco S. (2024). LOX-1 in Cardiovascular Disease: A Comprehensive Molecular and Clinical Review. Int J Mol Sci 25: PubMed PMID: 38791315.

14. Mosalmanzadeh N, Pence BD. (2024). Oxidized Low-Density Lipoprotein and Its Role in Immunometabolism. Int J Mol Sci 25: PubMed PMID: 39518939.

15. Sawyer L. (2021). beta-Lactoglobulin and Glycodelin: Two Sides of the Same Coin? Front Physiol 12: 678080. PubMed PMID: 34093238.

16. Qian S, He Y, Li R, Sun P, Zhang X, Pan L, Xu Z, Feng Z, Lian R, Yu L. (2025). Polymeric immunoglobulin receptor (pIgR) in cancer progression: a critical role and potential therapeutic target. Apoptosis: PubMed PMID: 40415061.

17. Lyu J, Jiang M, Zhu Z, Wu H, Kang H, Hao X, Cheng S, Guo H, Shen X, Wu T, Chang J, Wang C. (2024). Identification of biomarkers and potential therapeutic targets for pancreatic cancer by proteomic analysis in two prospective cohorts. Cell Genom 4: 100561. PubMed PMID: 38754433.

18. Kurowska P, Mlyczynska E, Dawid M, Jurek M, Klimczyk D, Dupont J, Rak A. (2021). Review: Vaspin (SERPINA12) Expression and Function in Endocrine Cells. Cells 10: PubMed PMID: 34359881.

19. Morris R, Butler L, Perkins A, Kershaw NJ, Babon JJ. (2021). The Role of LNK (SH2B3) in the Regulation of JAK-STAT Signalling in Haematopoiesis. Pharmaceuticals (Basel) 15: PubMed PMID: 35056081.

20. Ware CF, Croft M, Neil GA. (2022). Realigning the LIGHT signaling network to control dysregulated inflammation. J Exp Med 219: PubMed PMID: 35604387.

21. Lee WH, Kim SH, Lee Y, Lee BB, Kwon B, Song H, Kwon BS, Park JE. (2001). Tumor necrosis factor receptor superfamily 14 is involved in atherogenesis by inducing proinflammatory cytokines and matrix metalloproteinases. Arterioscler Thromb Vasc Biol 21: 2004-10. PubMed PMID: 11742877.

22. So T, Ishii N. (2019). The TNF-TNFR Family of Co-signal Molecules. Adv Exp Med Biol 1189: 53-84. PubMed PMID: 31758531.

23. Picca A, Nguyen NV, Calvani R, Dale M, Fredolini C, Marzetti E, Calderon-Larranaga A, Vetrano DL. (2025). Longitudinal changes in blood-borne geroscience biomarkers: results from a population-based study. Geroscience: PubMed PMID: 40272732.

24. Ren X, Shan WH, Wei LL, Gong CC, Pei DS. (2018). ACP5: Its Structure, Distribution, Regulation and Novel Functions. Anticancer Agents Med Chem 18: 1082-90. PubMed PMID: 29637867.

25. Okada D. (2024). Plasma proteins as potential biomarkers of aging of single tissue and cell type. Biogerontology 25: 177-81. PubMed PMID: 37707684.

26. Katagiri T, Tsukamoto S, Kuratani M. (2021). Accumulated Knowledge of Activin Receptor-Like Kinase 2 (ALK2)/Activin A Receptor, Type 1 (ACVR1) as a Target for Human Disorders. Biomedicines 9: PubMed PMID: 34206903.

27. van der Kraan PM. (2014). Age-related alterations in TGF beta signaling as a causal factor of cartilage degeneration in osteoarthritis. Biomed Mater Eng 24: 75-80. PubMed PMID: 24928920.

28. Sacco MA, Gualtieri S, Cordasco F, Tarallo AP, Verrina MC, Princi A, Bruni A, Garofalo E, Aquila I. (2024). The Role of Adrenomedullin as a Predictive Marker of the Risk of Death and Adverse Clinical Events: A Review of the Literature. J Clin Med 13: PubMed PMID: 39200990.

29. Larrayoz IM, Ferrero H, Martisova E, Gil-Bea FJ, Ramirez MJ, Martinez A. (2017). Adrenomedullin Contributes to Age-Related Memory Loss in Mice and Is Elevated in Aging Human Brains. Front Mol Neurosci 10: 384. PubMed PMID: 29187812.

30. Becker KL, Nylen ES, White JC, Muller B, Snider RH, Jr. (2004). Clinical review 167: Procalcitonin and the calcitonin gene family of peptides in inflammation, infection, and sepsis: a journey from calcitonin back to its precursors. J Clin Endocrinol Metab 89: 1512-25. PubMed PMID: 15070906.

31. Brigstock DR. (2003). The CCN family: a new stimulus package. J Endocrinol 178: 169-75. PubMed PMID: 12904165.

32. Zhang P, Jimenez SA, Stokes DG. (2003). Regulation of human COL9A1 gene expression. Activation of the proximal promoter region by SOX9. J Biol Chem 278: 117-23. PubMed PMID: 12399468.

33. Zeeuwen PL, Cheng T, Schalkwijk J. (2009). The biology of cystatin M/E and its cognate target proteases. J Invest Dermatol 129: 1327-38. PubMed PMID: 19262604.

34. Ohguro H, Watanabe M, Hikage F, Sato T, Nishikiori N, Umetsu A, Higashide M, Ogawa T, Furuhashi M. (2024). Fatty Acid-Binding Protein 4-Mediated Regulation Is Pivotally Involved in Retinal Pathophysiology: A Review. Int J Mol Sci 25: PubMed PMID: 39062961.

35. Saade M, Araujo de Souza G, Scavone C, Kinoshita PF. (2021). The Role of GPNMB in Inflammation. Front Immunol 12: 674739. PubMed PMID: 34054862.

36. Budge KM, Neal ML, Richardson JR, Safadi FF. (2018). Glycoprotein NMB: an Emerging Role in Neurodegenerative Disease. Mol Neurobiol 55: 5167-76. PubMed PMID: 28856541.

37. Costa Silva RCM, Correa LHT. (2022). Heme Oxygenase 1 in Vertebrates: Friend and Foe. Cell Biochem Biophys 80: 97-113. PubMed PMID: 34800278.

38. Schipper HM, Song W, Tavitian A, Cressatti M. (2019). The sinister face of heme oxygenase-1 in brain aging and disease. Prog Neurobiol 172: 40-70. PubMed PMID: 30009872.

39. Fernandes MB, Barata JT. (2023). IL-7 and IL-7R in health and disease: An update through COVID times. Adv Biol Regul 87: 100940. PubMed PMID: 36503870.

40. Passtoors WM, van den Akker EB, Deelen J, Maier AB, van der Breggen R, Jansen R, Trompet S, van Heemst D, Derhovanessian E, Pawelec G, van Ommen GJ, Slagboom PE, Beekman M. (2015). IL7R gene expression network associates with human healthy ageing. Immun Ageing 12: 21. PubMed PMID: 26566388.

41. Li H, Cui Y, Luan J, Zhang X, Li C, Zhou X, Shi L, Wang H, Han J. (2016). PRELP (proline/arginine-rich end leucine-rich repeat protein) promotes osteoblastic differentiation of preosteoblastic MC3T3-E1 cells by regulating the beta-catenin pathway. Biochem Biophys Res Commun 470: 558-62. PubMed PMID: 26809092.

42. Balasubramaniam M, Mokhtar AMA. (2024). Past and present discovery of the BAFF/APRIL system - A bibliometric study from 1999 to 2023. Cell Signal 120: 111201. PubMed PMID: 38714287.

43. Rossi M, Anerillas C, Idda ML, Munk R, Shin CH, Donega S, Tsitsipatis D, Herman AB, Martindale JL, Yang X, Piao Y, Mazan-Mamczarz K, Fan J, Ferrucci L, Johnson PF, De S, Abdelmohsen K, Gorospe M. (2023). Pleiotropic effects of BAFF on the senescence-associated secretome and growth arrest. Elife 12: PubMed PMID: 37083495.

44. Digre A, Lindskog C. (2021). The Human Protein Atlas-Spatial localization of the human proteome in health and disease. Protein Sci 30: 218-33. PubMed PMID: 33146890.
